# A highly efficient open-shell singlet luminescent diradical with strong magnetoluminescence properties

**DOI:** 10.1038/s41377-023-01314-z

**Published:** 2023-11-14

**Authors:** Alim Abdurahman, Li Shen, Jingmin Wang, Meiling Niu, Ping Li, Qiming Peng, Jianpu Wang, Geyu Lu

**Affiliations:** 1 State Key Laboratory of Integrated Optoelectronics, College of Electronic Science and Engineering, Jilin University, Qianjin Avenue 2699, Changchun, 130012 China; 2 College of Chemical Engineering and Environmental Chemistry, Weifang University, Weifang, 261061 China; 3 Key Laboratory of Flexible Electronics (KLOFE), Institute of Advanced Materials (IAM) & School of Flexible Electronics (Future Technologies), Nanjing Tech University (NanjingTech), 30 South Puzhu Road, Nanjing, 211816 China; 4 Key Laboratory for Organic Electronics and Information Displays & Institute of Advanced Materials (IAM) National Synergistic Innovation Center for Advanced Materials (SICAM), Nanjing University of Posts & Telecommunications, 9 Wenyuan Road, Nanjing, 210023 China

**Keywords:** Magneto-optics, Optical materials and structures

## Abstract

Developing open-shell singlet (OS) diradicals with high luminescent properties and exceptional single-molecule magnetoluminescence (ML) performance is extremely challenging. Herein, we propose a concept to enhance luminescent efficiency by adjusting the donor conjugation of OS diradicals, thereby achieving a highly luminescent diradical, DR1, with outstanding stability and making it a viable option for use in the emitting layer of organic light-emitting diodes (OLEDs). More importantly, the 0.5 wt%-DR1 doped film demonstrates significant single-molecule magnetoluminescence (ML) properties. A giant ML value of 210% is achieved at a magnetic field of 7 T, showing the great potential of DR1 in magneto-optoelectronic devices.

## Abstract (web summary)

A highly efficient open-shell singlet luminescent diradical demonstrates significant magnetoluminescence properties. A giant magnetoluminescence value of 210% is achieved at a magnetic field of 7 T, showing great potential in magneto-optoelectronic devices.

## Graphical abstract

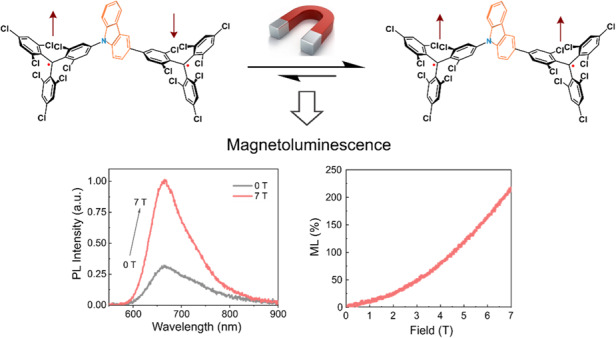

## Introduction

Open-shell singlet (OS) diradicals are important building blocks for functional molecular materials^1–18^, with a large number of pioneering works by researchers advancing their development and applications across various fields^19–40^. Despite this progress, there remains a lack of research regarding luminescent OS diradicals, hindering their potential use in optoelectronic applications. In fact, the luminescent diradicals are rare chemical species, there are only a few reports to date^41–44^. Recently, we reported the first luminescent Müller’s hydrocarbon with OS ground state^45^, but unfortunately, its photoluminescence quantum yield (PLQY) was found to be very low (0.4%), rendering it suitable only for conceptual exploration and hindering its practical applications in optoelectronic devices.

Magnetic field effects (MFEs) on the luminescence, i.e., magnetoluminescence (ML) of radicals, hold great promise for developing novel exciton spin manipulation methodologies that are unachievable by conventional closed-shell luminescent molecules^46–50^. In 2018, Kusamoto and co-workers first reported MLs in organic radical excimer species, which opened the gate to this field^46^. Recently, Kusamoto and co-workers elegantly designed and synthesized a spatially confined luminescent diradical and observed its single-molecule ML properties^50^. Nevertheless, the development of highly luminescent diradicals and the achievement of their efficient single-molecule ML properties continue to be a formidable challenge.

**Fig. 1:**
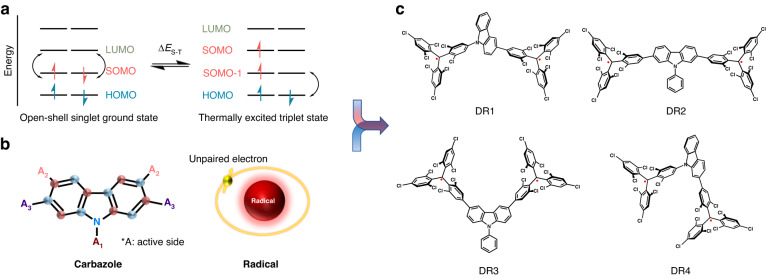
Strategy for molecular design. **a** Electronic configuration of the ground state for open-shell singlet (OS) diradicals. **b** Luminescent diradical design strategy for carbazole bridges. **c** Chemical structures of designed diradicals DR1–DR4

Based on the first-principle calculations, we find that for an OS diradical, the radiative decay of singlet excitons corresponds to electronic transitions between the singly occupied molecular orbital (SOMO) and the lowest unoccupied molecular orbital (LUMO). However, due to the electron correlation, the electron density distributions of the SOMO and LUMO for α and β electrons located in the different fragments (all of which are located on the two radical centers) are cross-distributed (Fig. S1). This makes the transition out-of-phase with minimal oscillator strength since, in the zero-order approximation, electronic transitions can only occur between orbitals of the same spin (Fig. 1a). In contrast, the radiative decay of triplet excitons involves the highest occupied molecular orbital (HOMO) of the donor part and the SOMO of radical center (Fig. 1a and Fig. S1). Therefore, elegantly adjusting the donor conjugation of the diradical can improve its transition oscillator strength and enhance its luminescence properties.

Following the theoretical framework above, we select carbazole, a mild donor unit with a non-alternating molecular structure and multiple active sites as the bridging group, and tris(2,4,6-trichlorophenyl)methyl (TTM) as the radical center to design four diradicals, DR1–DR4 (Fig. 1b, c). To identify the optimal candidate, we conducted a series of theoretical investigations on the four molecules. Using unrestricted density functional theory (UDFT) and time-dependent UDFT at the B3LYP/6-31 G(d,p) level, we obtain the ground-state and excited-state properties. The calculated singlet-triplet energy gap (Δ*E*_S-T_) of DR1–DR4 is −0.022, −0.031, −0.008, and 0.004 kcal mol^−1^, respectively, indicating weak interactions between the two TTM mono-radical centers and revealing that DR1–DR3 are OS diradicals (Table S1). The negligible differences observed in spin density distributions and the optimized geometries of OS and thermally excited triplet (T_t_) (Figs. S2–S5) are consistent with the small Δ*E*_S-T_. The diradical character index, y_0_, was widely used to describe the degree of diradical character, ranging from 0 for a closed-shell (CS) electronic structure to 1 for a pure diradical^8^. DR1–DR4 show large *y*_0_ values of >0.90, revealing excellent diradical features of the four molecules (Table S1). However, the quantum chemical calculations reveal that the different connection modes between the bridging carbazole unit and the two TTM mono-radicals largely affect their interaction strength, leading to different photophysical properties for the four molecules. The calculation results indicate that DR1 exhibits the most efficient electron-hole separation and higher oscillator strength for the first excited state transitions than the other three diradicals (Figs. S7, S8 and Tables S2, S3).

## Results

### Synthesis and structure

**Scheme 1:**
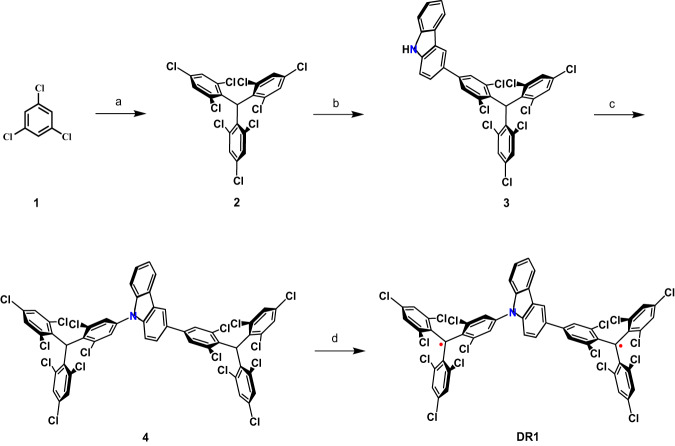
Synthetic route to DR1, Reagents and conditions: **a** CHCl_3_, AlCl_3_, heated at 70 °C for 3 h in a glass pressure vessel; **b** 3-(4,4,5,5-tetramethyl-1,3,2-dioxaborolan-2-yl)-carbazole, Pd(PPh_3_)_4_, K_3_PO_4_, toluene, ethanol, H_2_O, 95 °C, reflux for 48 h; **c** TTM, Cs_2_CO_3_, DMF, Dark, 160 °C, reflux for 6 h; **d** Anhydrous THF, t-BuOK, stirring for 12 h at room temperature, and then adding tetrachloro-p-quinone, stirring for 3 h

**Fig. 2:**
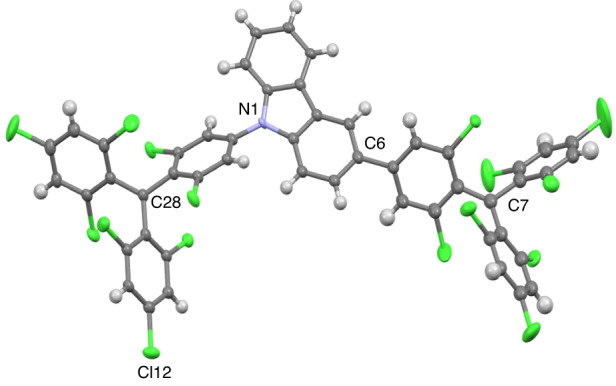
X-ray crystallographic structure of DR1

According to the theoretical considerations described above, we report herein a highly efficient OS luminescent diradical DR1, which was prepared in four steps from commercially available reagents (Scheme 1 and Supporting Information S2). To determine the molecular structure, a single crystal of the DR1 was obtained by slow evaporation from a methanol/dichloromethane solution at room temperature. The structure was then determined by synchrotron radiation (Fig. 2). As can be seen, the two triphenyl groups attached to N1 and C6 are propeller-shaped due to the steric repulsion of the chlorine atoms, while the sp^2^ hybridized carbon atoms C7 and C28 are the two unpaired radicals. The magnetic property of DR1 was investigated using a superconducting quantum interference device (SQUID). As shown in Fig. S9, the value of *χ*_*m*_*T* increases rapidly with increasing temperature from 2 to 100 K, which is typical for an open-shell singlet ground state in thermal equilibrium with a triplet state^51^. Fitting the *χ*_*m*_*T–T* curve with the Bleany–Bowers equation^52^, we obtain a Δ*E*_S-T_ of −0.051 kcal mol^−1^, which is consistent with the theoretically calculated value. Moreover, DR1 exhibits a single-line electron paramagnetic resonance (EPR) signal in the Δ*m*_*s*_ = ±1 region, and a signal corresponding to the Δ*m*_*s*_ = ±2 transitions is also observed at 77 K (Fig. S10).

### Photophysical and electroluminescent properties

**Fig. 3:**
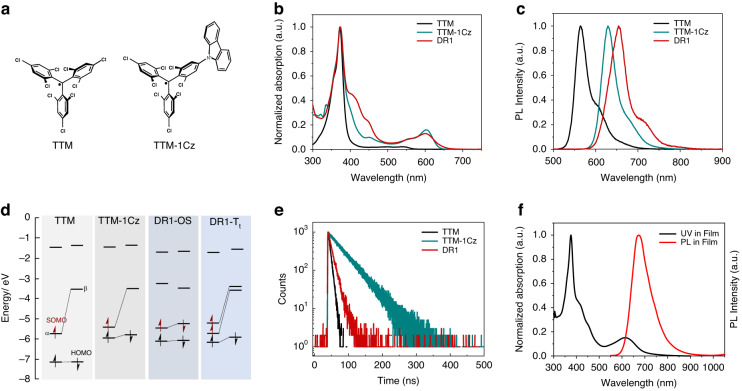
The photophysical properties of radicals. **a** Chemical structures of the mono-radical TTM and TTM-1Cz. **b** Normalized UV/Vis absorption. **c** PL spectra and **e** transient photoluminescence decay spectra (excitation at 375 nm) of TTM, TTM-1Cz, and DR1 in cyclohexane solution at room temperature. **d** Energy diagrams of the TTM, TTM-1Cz, and DR1 (in open-shell singlet (OS) ground state and thermally excited triplet (T_t_) state) calculated by UDFT (UB3LYP/6-31G(d,p)). **f** Normalized absorption and PL spectra of DR1 (0.5 wt%) doped in PMMA film at room temperature

To evaluate the luminescent properties of DR1, a series of photophysical studies were conducted on it and its two fragments (mono-radical TTM^53^ and ((4-(N-carbazolyl)-2,6-dichlorophenyl) bis(2,4,6-trichlorophenyl) methyl (TTM-1Cz)^54,55^; Fig. 3a) at room temperature. Figure 3b, c shows the normalized UV/Vis absorption, photoluminescence (PL), and transient PL decay spectra of mono-radicals and DR1 in cyclohexane. These three radicals show strong absorption bands at 375 nm, attributed to the transition of electrons on α-SOMO to higher energy levels. Compared to the weak absorption band of TTM at long wavelength (~540 nm), there is a relatively strong absorption band at ~600 nm in TTM-1Cz and DR1, which is attributed to the transition of electrons on β-HOMO to β-SOMO (Fig. 3d, see Supporting Information for details). The emission band of DR1 (654 nm) exhibits a significant red shift compared to TTM (564 nm) and TTM-1Cz (628 nm). The PLQY of TTM, TTM-1Cz, and DR1 are 2.0%, 53.0%, and 16.0%, respectively. Notably, the PLQY of DR1 is 40 times higher than that of the firstly reported luminescent Müller hydrocarbon TTM-PhTTM (0.4%)^47^. The PL decay lifetime (*τ*) of DR1 (10.6 ns) is between those of TTM (5.6 ns) and TTM-1Cz (41.3 ns) (Fig. 3e). Then, the radiative and non-radiative rate constant (*k*_*r*_ and *k*_*nr*_) of DR1 are estimated to be 1.51 × 10^7^ and 7.92 × 10^7^ s^−1^, respectively. Notably, the *k*_*r*_ of DR1 is higher than those of TTM (0.35 × 10^7^ s^−^^1^) and TTM-1Cz (1.28 × 10^7^ s^−1^), which is due to the higher transition oscillator strength of DR1 (Table S2). While the *k*_*nr*_ of DR1 is lower than that of TTM (17.5 × 10^7^ s^−1^) and higher than that of TTM-1Cz (1.13 × 10^7^ s^−1^). The larger *k*_*nr*_ of DR1 than TTM-1Cz may be due to its smaller energy gap and the intramolecular spin interaction, which can accelerate the internal conversion to the ground state.

To investigate the ground and excited state characteristics of DR1, we conducted measurements of UV/vis absorption, PL, *τ* and PLQY in various solvents with different polarities (Fig. S11 and Table S5). As the solvent polarity increases, the UV/vis absorptions of DR1 remain largely constant, indicating minimal dipolar changes in the ground state across different polar solvents. On the contrary, the emission spectra exhibit an obvious bathochromic shift; the PLQY and *τ* start with a slight increase followed by a decrease with increasing solvent polarity, showing a parabolic trend. The highest PLQY reaches 25% in toluene, while in high-polarity chloroform, it remains 19%, four times higher than that of TTM-1Cz (5%). Overall, these photophysical properties indicate that the excited state of DR1 is a hybridized local and charge-transfer (CT) state, as compared with the local excited state of TTM and CT-dominated excited state of TTM-1Cz^56,57^.

The DR1 doped (0.5 wt% in PMMA) film shows identical UV/Vis absorption to that in solution while exhibiting a deep-red emission (673 nm) with a PLQY of 14.1% (Fig. 3f). We found that at low doping concentrations of DR1 (0.1–2 wt%), the emission does not exhibit significant red shift or broadening, indicating that the luminescence is primarily contributed by single molecules. However, at higher concentrations (10–30 wt%), the emission shows significant redshifts (to ~690 nm) and broadening, while PLQYs decrease, suggesting the possible occurrence of aggregation and excimer formation (Fig. S13 and Table S6). Fig. S14 shows the temperature-dependent PL of the 0.5 wt%-DR1 doped film. As the temperature decreases from 300 to 100 K (Fig. S14a), intermolecular vibrations weaken, resulting in a decrease in the rate of thermal radiation recombination and an increase in the PL intensity. This behavior is similar to that of the mono-radical TTM-1Cz (Fig. S15a). However, as the temperature decreases further from 100 to 2 K (Fig. S14b), the PL intensity decreases, revealing that singlet excitons are less emissive than triplet excitons for DR1, which is consistent with the quantum chemical calculations (Table S2). When the temperature falls below 20 K, the PL rapidly weakens, indicating a much-increased occupation of the OS ground state at low temperatures due to its small Δ*E*_S-T_. This is in contrast to the behavior of TTM-1Cz, where the PL intensity slightly increases with temperature from 100 to 2 K (Fig. S15b) because TTM-1Cz is a doublet molecule, and the spin-statistics is not temperature-dependent. The temperature-dependent PL of 20 wt%-DR1 doped film (Fig. S16) is similar to that of the 0.5 wt%-DR1. However, as the temperature decreases from 40 to 2 K, the excimer emission band (peaks at 820 nm) gradually becomes dominant, and the intensity remains unchanged.

DR1 shows excellent thermal- and photo-stabilities. As can be seen in Fig. S17a, the thermogravimetric analysis (TGA) indicates that DR1 has a high thermal decomposition temperature up to 346 °C in a nitrogen atmosphere. The photo-stability of DR1 was measured in cyclohexane and compared with mono-radicals TTM and TTM-1Cz. The estimated half-lifetime of DR1 (3.7 × 10^4^ s) is 1500 and 25 times higher than that of TTM (2.4 × 10^1^ s) and TTM-1Cz (1.5 × 10^3^ s), respectively (Fig. S17b). We also carried out a theoretical analysis of the thermodynamic and kinetic stability^58^ of DR1 and compared it with mono-radicals (Table S7 and Fig. S18). The calculated results are consistent with our experimental observations.

The greatly enhanced PLQY and excellent stability of DR1 inspired us to explore its potential as the emitting layer in organic light-emitting diodes (OLEDs). Accordingly, we fabricated solution-processed OLEDs utilizing DR1 (0.5 wt%) as the emissive dopant (see details in Supporting Information). The OLEDs exhibit deep-red emission peaking at 680 nm with the maximum external quantum efficiency (EQE)^59^ of ~1.0% (Fig. S19).

### ML properties

**Fig. 4:**
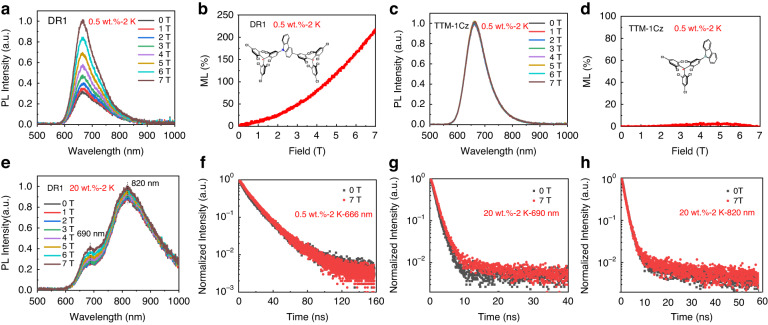
The ML characteristics at 2 K for DR1 (0.5 wt% and 20 wt%) and mono-radical TTM-1Cz (0.5 wt%) doped in PMMA films. **a** PL spectra of DR1 (0.5 wt%) at different magnetic fields. **b** ML of DR1 (0.5 wt%). **c** PL spectra of TTM-1Cz (0.5 wt%) at different magnetic fields. **d** ML of TTM-1Cz. **e** PL spectra of DR1 (20 wt%) at different magnetic fields. **f** The transient PL decay spectra of DR1 (0.5 wt%) with and without a magnetic field of 7 T. **g** The transient PL decay spectra of DR1 (20 wt%) at 690 nm with and without a magnetic field of 7 T. **h** The transient PL decay spectra of DR1 (20 wt%) at 820 nm with and without a magnetic field of 7 T

It is interesting to find that DR1 shows quite strong ML properties. Figure 4a shows that the PL intensity of DR1 single-molecule (0.5 wt.%) significantly enhances with the increase in magnetic field (from 0 to 7 T) at 2 K, achieving a giant ML value of 210% at 7 T (Fig. 4b). In contrast, the mono-radical TTM-1Cz (0.5 wt%) exhibits almost no ML effect (Fig. 4c). This indicates that the interaction between two electrons within a single-molecule of DR1 plays a key role for MLs. For the high concentration (20 wt%) doped film, both the aggregation-induced red-shifted single-molecule emission and excimer emission increase with magnetic field, and the ML value of aggregated single-molecule emission and excimer emission are 50% and 7.5%, respectively (Figs. S23 and. S24), similar to the previously reported ML in high concentration mono-radical (10 wt%)^46^.

To understand the ML properties of DR1, we carried out transient PL measurements. As can be seen from Fig. 4f, the decay processes of DR1 single-molecule (0.5 wt%) emission do not change when the magnetic field is applied, revealing that the magnetic field cannot influence the transition processes of the excitons. It is likely to change the statistics of excitons with different spin configurations, i.e., changes in the spin states of electrons. The transient PL with and without a magnetic field of 7 T for high-concentration doped DR1 (20 wt%) are presented in Fig. 4g, h. While the excimer decay process does not change with the external magnetic field (Fig. 4h), the decay process of aggregated single-molecular emission does show a response to the magnetic field (Fig. 4g). This indicates that the magnetic field can weaken the intermolecular interactions by control the spins of electrons since frontier orbital electrons with same spins tend to repel each other^60–62^. Therefore, the aggregation effects decreased, leading to a decreased non-radiative recombination rate and increased lifetime for the emission of aggregated single molecules. In addition, temperature-dependent ML tests were conducted on two films, as shown in Fig. 4b and Figs. S21 and S22. The MLs of both 0.5 wt% and 20 wt% doped DR1 decrease with temperature and vanish at ~100 K. This means that the magnetic field is more likely to affect the electron spin rather than the intersystem crossing as proposed previously^46,48^. Otherwise, significant MLs can be observed at high temperatures.

**Fig. 5:**
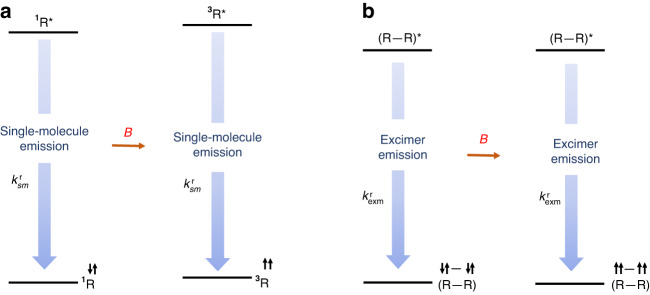
Magnetoluminescence mechanism of DR1 at low temperature. **a** single-molecule. **b** Excimer molecule. (^1^R, ^3^R: singlet and triplet of single-molecule in the ground state; ^1^R*, ^3^R*: singlet and triplet of single-molecule in the excited state; (R–R): excimer molecule; ${{\boldsymbol{k}}}_{{\boldsymbol{sm}}}^{{\boldsymbol{r}}}$: single-molecule radiation transition; ${{\boldsymbol{k}}}_{{\boldsymbol{exm}}}^{{\boldsymbol{r}}}$: excimer molecule radiative transition)

Based on these findings, we propose a possible mechanism for ML in DR1 (Fig. 5) based on magnetic field-induced spin polarization^63^. As shown in Fig. 5a, for DR1 single-molecular emission, the molecules tend to occupy the OS ground states as the temperature decreases. Therefore, at low temperatures, the number of less-emissive singlet excitons is significantly higher than that of high-emissive triplet excitons generated by photoexcitation. This is primarily due to the lower occupation of the T_t_ ground state and the weak thermally forbidden intersystem crossing from singlet excitons to triplet excitons, leading to the decreased PL intensity as the temperature decreases (Fig. S14b). When the magnetic field is applied, electron spins tend to be aligned, leading to the increased occupation of the T_t_ ground state and triplet excitons and, thus, increased PL, i.e., the ML. We note that this model is in line with temperature-dependent MLs. The ML mechanism of high-concentration doped DR1 is similar to that of single-molecular emission. For the aggregated single-molecular emission, we should point out that the aggregation of the molecules plays an important role. Since the aggregation can highly annihilate the PL emission (mostly from triplet excitons), one can expect a decreased ML. For the excimer emission, since the decay processes do not change with the magnetic field, we can conclude that the magnetic field cannot influence the transition processes of the excimers. A probable mechanism for the ML of DR1 excimer emission should come from the difference in PL efficiencies between excimers formed by triplets and those formed by singlets. Therefore, an increased number of triplet excimers can lead to an increased total excimer emission intensity. However, the significantly decreased ML of the excimer emission than that of the single-molecular emission indicates that the PL efficiency of excimers formed by singlet excitons is close to that formed by triplet excitons, which is consistent with the temperature-insensitive excimer emission intensity (Fig. S16b). We note that this model is different from the one proposed by Kusamoto et al. ^47–50^, where the ML of single-molecular and excimer emissions from mono-radicals were clearly interpreted. Nevertheless, the underlying mechanism is the magnetic field-induced spin polarization (Fig. 5b), in line with that proposed by Kusamoto^50^.

## Discussion

We have proposed a concept to enhance the luminescence of OS diradicals, leading to the design of a new luminescent diradical, DR1. Both theoretical and experimental investigations confirm that DR1 is an OS in the ground state, with the ability to be thermally excited to the triplet state due to the small Δ*E*_S-T_. DR1 exhibits outstanding properties, including a PLQY that is 60 times higher than that of the luminescent Müller hydrocarbon, as well as excellent thermal- and photo-stability. Furthermore, we have successfully fabricated deep-red OLEDs based on DR1. Moreover, at 7 T and 2 K, a giant single-molecule ML of 210% was achieved, highlighting the strong magneto-optical properties of DR1. Our study represents a significant step towards the application of OS luminescent diradicals in magneto-optoelectronic fields.

## Materials and methods

### Materials

All chemical agents and solvents, unless otherwise stated, were purchased from commercial suppliers and used directly without further purification. The intermediate 2(HTTM), mono-radical TTM, and TTM-1Cz were prepared according to our previous reports^54,56^. DR1 was prepared in four steps from commercially available reagents (Supporting Information S2), and its crystallographic data (CCDC 2252807) are provided free of charge by the joint Cambridge Crystallographic Data Center and Fachinformationszentrum Karlsruhe Access Structures service.

### General characterization

The ^1^H nuclear magnetic resonance (NMR) spectra were recorded in (Methyl sulfoxide)-d6 (d6-DMSO) on a Bruker Avance-III 500 NMR spectrometer at ambient temperature. GC–MS mass spectra were recorded on a Thermo Fisher ITQ1100 mass spectrometer. MALDI-TOF mass spectra were recorded on a Bruker Autoflex speed TOF/TOF mass spectrometer with DCTB as a matrix. EPR spectra were recorded on a Bruker ELEXSYS-II E500 CW-EPR spectrometer. Thermal gravimetric analysis (TGA) curves were obtained on the Pyris1 TGA thermal analysis system at a heating rate of 20 °C min^−1^ in a nitrogen atmosphere. Elemental analysis was conducted by an Elementar vario MICRO cube instrument. Single crystal X-ray diffraction data of DR1 was collected using a synchrotron X-ray source at the Shanghai Synchrotron Radiation Facility. The crystal structure was determined by direct methods and further refined by the full matrix least squares method of F2 using the SHELX-97 and Olex-2. Magnetic measurements were performed on a Quantum Design 6.5 Tesla SQUID-VSM system with a temperature range of 2–300 K and an applied field of 1000 Oe. A powder sample of DR1 with a weight of 5–10 mg was sealed in a plastic capsule. The magnetic moment was measured in the temperature range of 2–300 K. After correction of diamagnetic contributions from the sample, using tabulated constants, sample holder, and paramagnetic contamination, the magnetic data were fitted with Bleaney–Bowers equation^52^.

### Theoretical calculations

All calculations were performed with the Gaussian 16 program package. The geometries of all compounds were optimized as open-shell (OS) singlets by the spin-unrestricted broken-symmetry (BS) approach at the UB3LYP/6-31 G** theoretical level. This approach has been shown to provide reliable geometries and energies for singlet-state diradicals. Then these compounds were optimized as closed-shell (CS) singlets and thermally excited triplet (T_t_) states at the (U)B3LYP/6-31 G** level, respectively. All optimized geometries were confirmed to be local minima by vibrational analysis. The OS structures were shown to be stable structures. Δ*E*(OS-CS) and Δ*E*(OS-T_t_) were calculated as the energy differences between OS structure and CS structure, T_t_ structure, respectively.

### Photo-physics

Ultraviolet-visible (UV–Vis) and photoluminescence (PL) spectra of the radicals were recorded on a Shimadzu UV-2550 spectrophotometer and a Shimadzu 5301PC spectrophotometer. The intensity of luminescence at 654 nm (for DR1), 564 nm (for TTM), and 628 nm (for TTM-1Cz) were monitored exciting at 370 nm light (excitation slit was 20 nm, and shutter control was off). The relative PLQYs were measured using a Shimadzu UV-2550 spectrophotometer and Edinburgh fluorescence spectrometer (FLS980). The fluorescence lifetimes were measured with FLS980. The temperature-dependent PL spectra of the radicals were measured using a spectrometer (Ocean Optics QE65 Pro), and a Spectromag PT liquid helium-free superconducting magneto-optical system (Oxford Instruments NanoScience) was used to provide different temperatures with 2 ~ 300 K and magnetic fields from 0 to 7 T.

### Supplementary information

Supporting Information
