# Supplementary material for: A highly efficient open-shell singlet luminescent diradical with strong magnetoluminescence properties
Source: Light Sci Appl. 2023 Nov 14;12:272. doi: 10.1038/s41377-023-01314-z (PMC10645991; doi:10.1038/s41377-023-01314-z)
Supplement: Supplementary file 1 — Supporting Information [file 41377_2023_1314_MOESM1_ESM.docx]

Supporting Information

**A Highly Efficient Open-shell Singlet Luminescent Diradical with Strong Magnetoluminescence Properties**

Alim Abdurahman,^†[1]^* Li Shen,^†[2]^ Jingmin Wang,^†[3]^ Meiling Niu,^[3]^ Ping Li,^[4]^ Qiming Peng,^[3]^* Jianpu Wang,^[3]^ Geyu Lu^[1]^*

*^1^* *State Key Laboratory of Integrated Optoelectronics, College of Electronic Science and Engineering, Jilin University, Qianjin Avenue 2699, Changchun, 130012, China*

*^2^* *College of Chemical Engineering and Environmental Chemistry, Weifang University, Weifang, 261061, China*

*^3^ Key Laboratory of Flexible Electronics (KLOFE)，Institute of Advanced Materials (IAM) & School of Flexible Electronics (Future Technologies), Nanjing Tech University (NanjingTech), 30 South Puzhu Road, Nanjing, 211816, China*

*^4^* *Key Laboratory for Organic Electronics and Information Displays & Institute of Advanced Materials (IAM) National Synergistic Innovation Center for Advanced Materials (SICAM)*

*Nanjing University of Posts & Telecommunications, 9 Wenyuan Road**, Nanjing, 210023, China*

*† These authors contributed equally to this work.*

** Authors to whom correspondence should be addressed;*

*alim@jlu.edu.cn (*Alim Abdurahman*); iamqmpeng@njtech.edu.cn* *(*Qiming Peng*); Lugy@jlu.edu.cn (*Geyu Lu*)*

**Contents**

S1. Theoretical calculations of radicals

S2. Material synthesis and characterisation

S3. Crystallographic data of DR1

S4. Magnetic susceptibility measurements of DR1

S5. Electron paramagnetic resonance (EPR) spectra of DR1

S6. Summary of photophysical properties for TTM, TTM-1Cz and DR1 in different solvents

S7. Excitation and emission spectrum of DR1 in cyclohexane

S8. The influence of doping concentration and temperature of DR1 (in PMMA) on photoluminescence

S9. Stabilitiy of TTM, TTM-1Cz and DR1

S10. Electroluminescence properties of DR1-based OLED

S11. ML properties of DR1 and TTM-1Cz.

**S1. Theoretical calculations of radicals**

**Computational details**

All these calculations were performed with the Gaussian 16 program package.^[1]^ The geometries of all compounds were optimized as open-shell (OS) singlets by the spin- unrestricted broken-symmetry (BS) approach at the UB3LYP/6-31G**^[2-3]^ theoretical level. This approach has been shown to provide reliable geometries and energies for singlet-state diradicals. Then these compounds were optimized as closed-shell (CS) singlets and thermally excited triplet (T_t_) states at the (U)B3LYP/6-31G** level, respectively. All optimized geometries were confirmed to be local minima by vibrational analysis. The OS structures were shown to be the stable structures. Δ*E*(OS-CS) and Δ*E*(OS-T_t_) were calculated as the energy differences between OS structure and CS structure, T_t_ structure, respectively.

Diradical character were determined using two different descriptors: (1) diradical character y_0_. It ranges from *y*_0_ = 0 (pure closed-shell) to *y*_0_ =1 (pure diradical), and calculated from the occupation numbers of natural orbitals using the spin-projected B3LYP/6-31G** level of theory as proposed by Yamaguchi.^[4]^ The parameters are obtained by the following equation:

$$y_{0}=1-\frac{2T_{i}}{1+T_{i}^{2}} (1)$$

Where *T*_i_ represents the orbital overlap between the corresponding orbitals. It is expressed in the terms of the occupation numbers (n_i_) of the UB3LYP/6-31G** natural orbitals (UNO) as

$$T_{i}=\frac{n_{HONO-i}-n_{LUNO+i}}{2} (2)$$

and *i*= 0, 1, 2 …….. (2) the Fractional occupation number weighted density (FOD) analysis was used as a quantitate description of the open-shell singlet diradical character of these compounds. The number of hot electrons, *N*^FOD^ were calculated at the FT-B3LYP/6-31G** level at a default electronic temperature (Tel) of 9000 K. The calculations were performed using the ORCA 5.0.3 program.^[5]^

Vertical electronic excitations were computed by using the time-dependent DFT (TD-DFT) approach. The excitation energies were calculated at UB3LYP/6-31G** level by using the OS and T_t_ geometries. In addition, the excitation characters were analysed with hole-electron analysis ^[6]^ by Multiwfn program,^[7]^ and the corresponding results were visualized by VMD programs.^[8]^ The SF-TDDFT calculations were performed at the theoretical level of BHandHLYP/def2-SVP using the ORCA 5.0.3.^[9-11]^ The corresponding auxiliary basis set def2/J has been also used for Coulomb fitting in a resolution-of-identity/chain-of-spheres (RIJCOSX) framework.

**Table S1.** Diradical character y_0_, Δ*E*_OS-Tt_ and Δ*E*_OS-CS_ of the four designed diradicals.

| Diradical | *y*_0_ | *N*^FOD^ | Δ*E*_OS-Tt_ (UDFT) | Δ*E*_OS-CS_ (UDFT) | Δ*E*_OS-Tt_ (SF-TDDFT) |
| --- | --- | --- | --- | --- | --- |
| DR1 | 0.947 | 3.50 | -0.022 | -20.39 | -0.082 |
| DR2 | 0.948 | 3.67 | -0.030 | -20.64 | -0.041 |
| DR3 | 0.972 | 3.67 | -0.008 | -21.58 | -0.046 |
| DR4 | 0.990 | 3.53 | 0.004 | -21.88 | 0.148 |

**
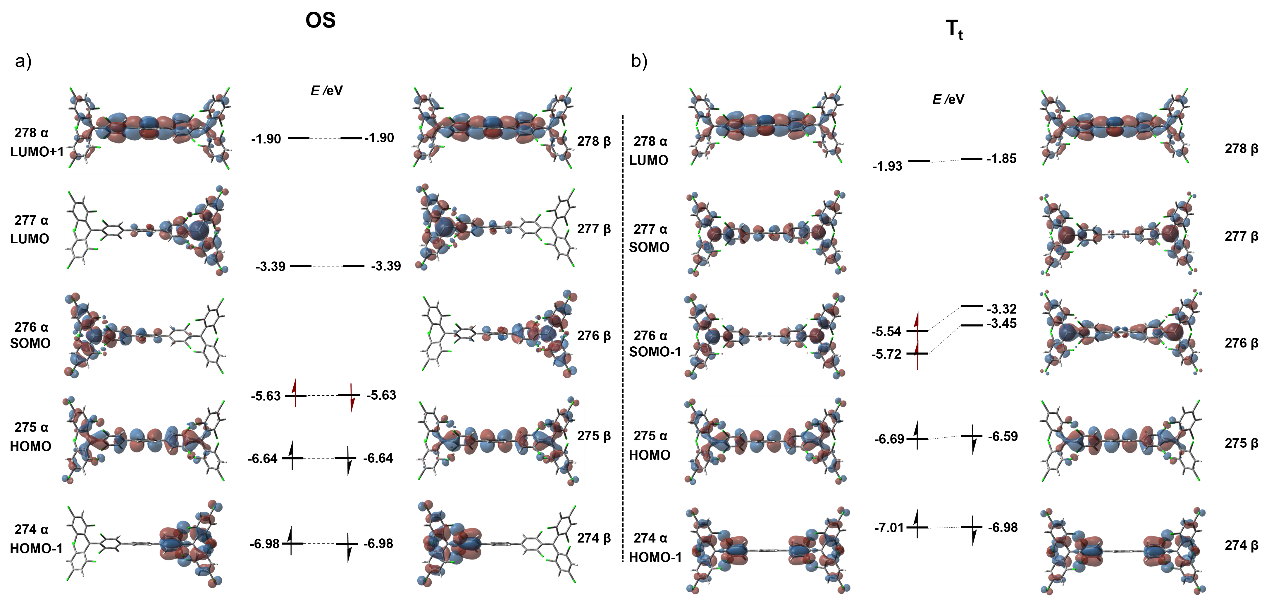
**

**Fig. S1** Frontier orbitals of previously reported luminescent diradical TTM-PhTTM^12^ in open-shell singlet (OS) ground state a) and thermally excited triplet (T_t_) state b) calculated by DFT methods (UB3LYP/6-31G (d,p)).


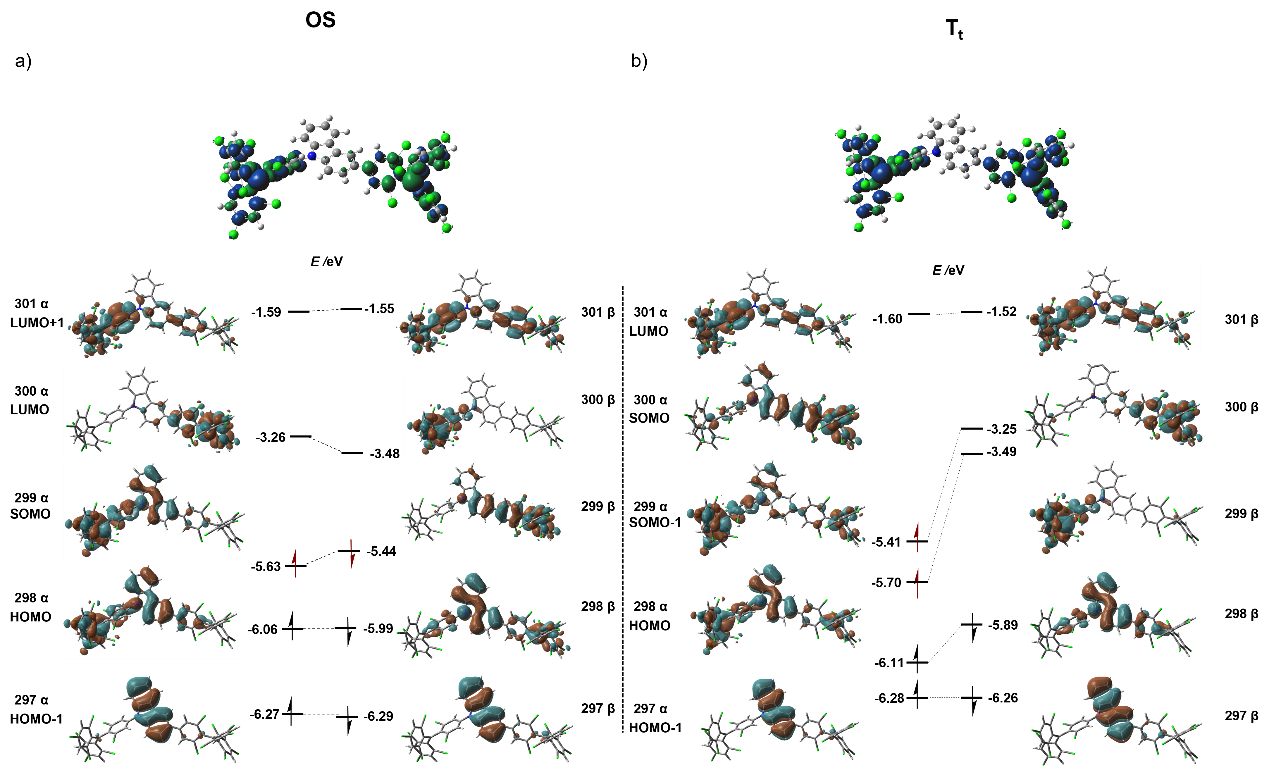


**Fig. S2** Spin density distributions (isovalue 0.0015) (up panels), Frontier orbitals (down panels) of DR1 in open-shell singlet (OS) ground state a) and thermally excited triplet (T_t_) state b) calculated by DFT methods (UB3LYP/6-31G (d,p)).


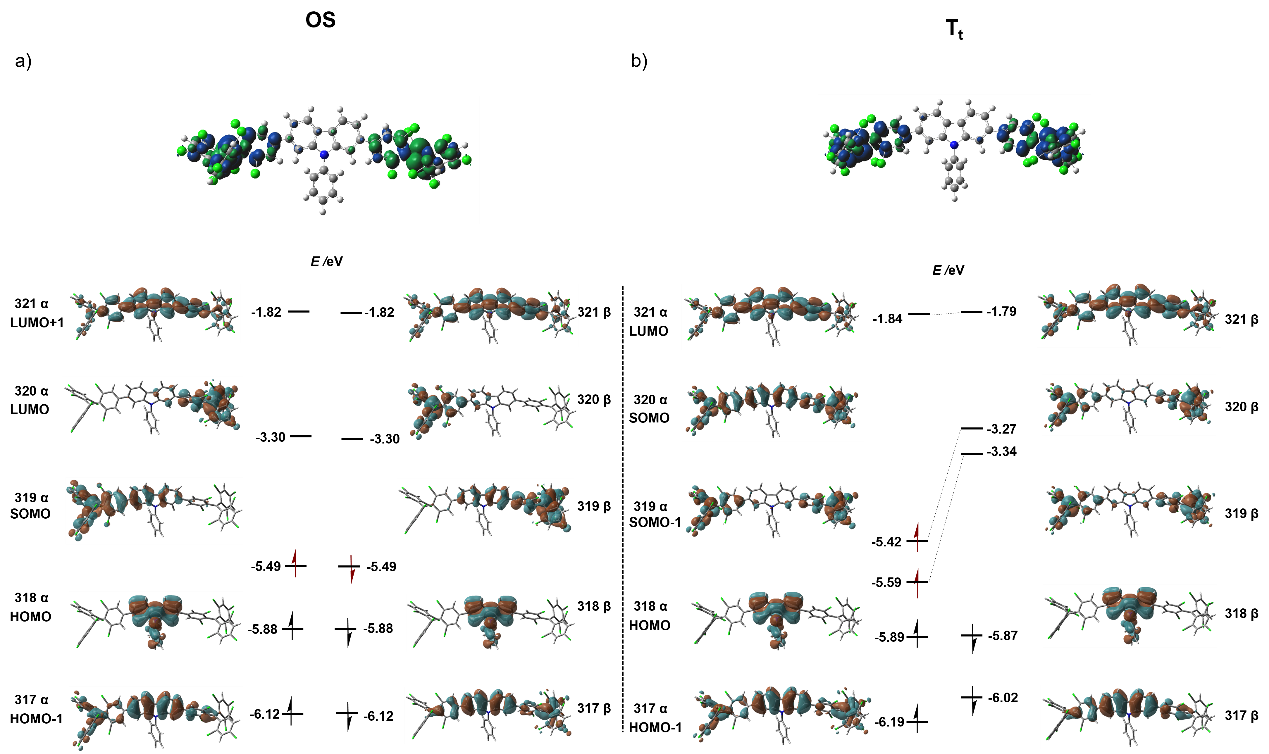


**Fig. S3** Spin density distributions (isovalue 0.0015) (up panels), Frontier orbitals (down panels) of DR2 in open-shell singlet (OS) ground state a) and thermally excited triplet (T_t_) state b) calculated by DFT methods (UB3LYP/6-31G (d,p)).


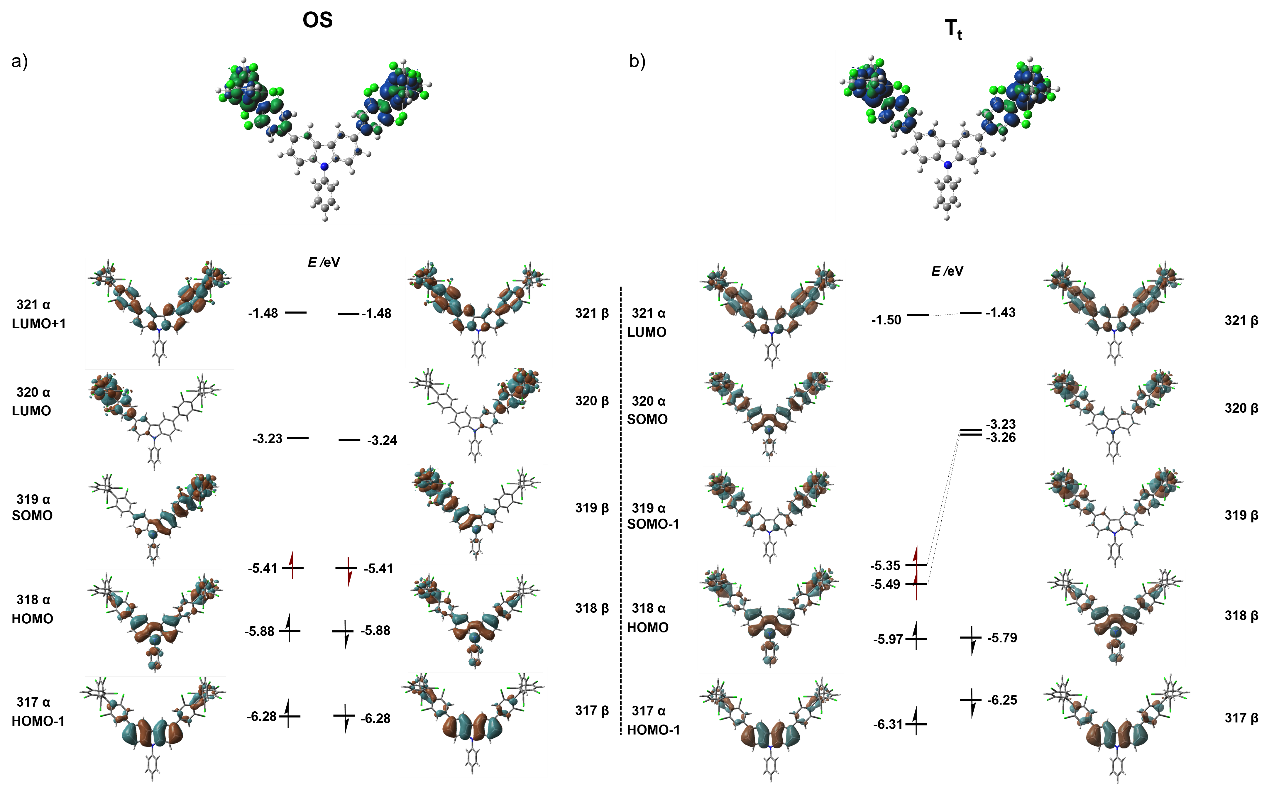


**Fig. S4** Spin density distributions (isovalue 0.0015) (up panels), Frontier orbitals (down panels) of DR3 in open-shell singlet (OS) ground state a) and thermally excited triplet (T_t_) state b) calculated by DFT methods (UB3LYP/6-31G (d,p)).


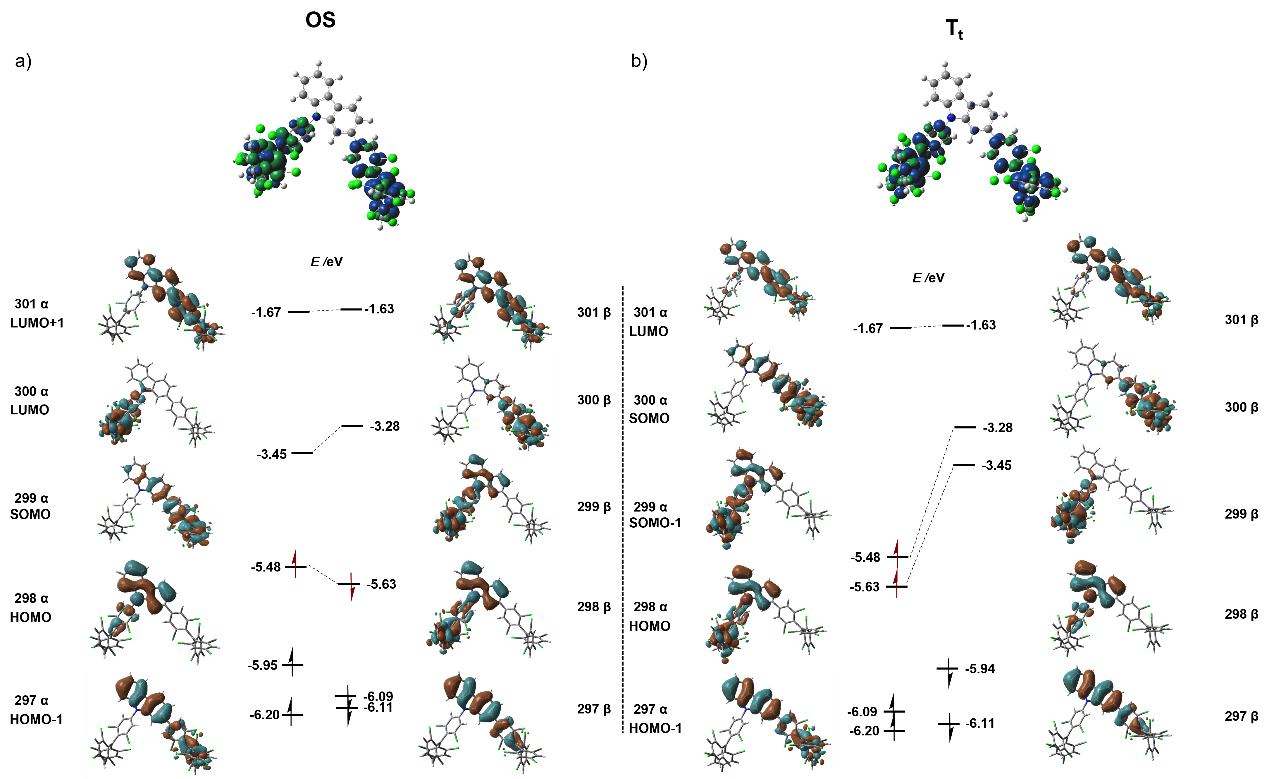


**Fig. S5** Spin density distributions (isovalue 0.0015) (up panels), Frontier orbitals (down panels) of DR4 in open-shell singlet (OS) ground state a) and thermally excited triplet (T_t_) state b) calculated by DFT methods (UB3LYP/6-31G (d,p)).


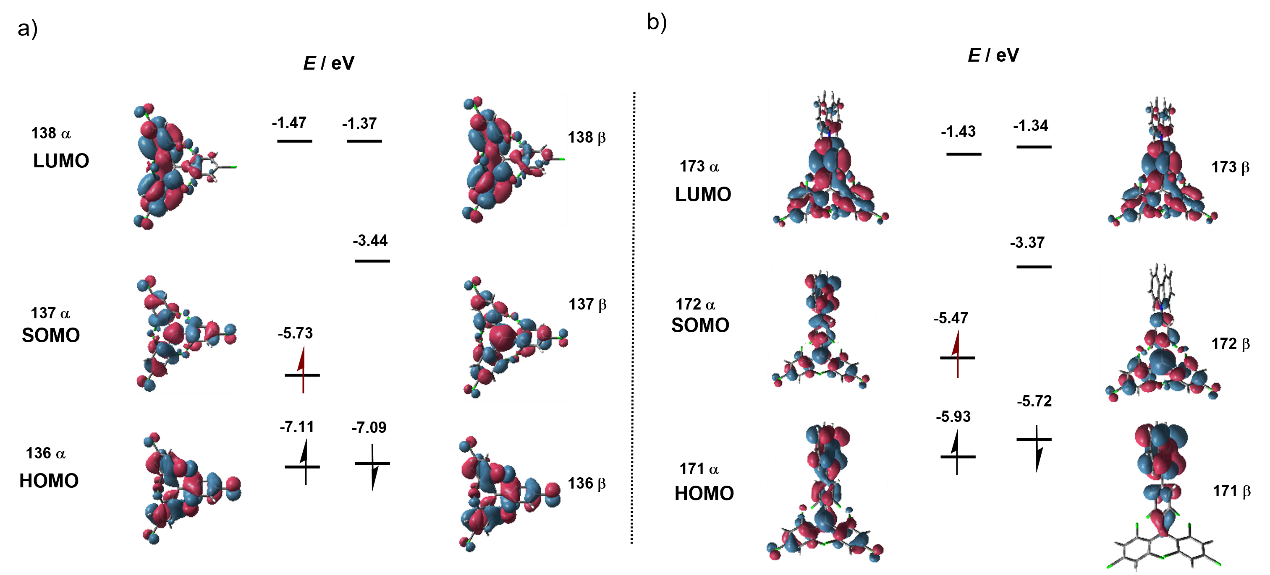


**Fig. S****6** Frontier orbitals of TTM (a) and TTM-1Cz (b) calculated by DFT methods (UB3LYP/6-31G (d,p)).

**Table S2.** Summary of selected calculated excitation energies, oscillator strength and contributions of major electronic transitions of mono-radical and diradicals.

|  | Transition | Wavelength (nm) | Energry/  (eV) | Osc.Strength (ƒ) | Major Contribution |
| --- | --- | --- | --- | --- | --- |
| TTM | D_0_→D | 466.95 | 2.6552 | 0.0223 | 136 B →137 B |
| TTM-1Cz | D_0_→D | 662.12 | 1.8725 | 0.0830 | 171 B→172 B |
| DR1 | OS | 737.73 | 1.6806 | 0.0510 | 299 B→ 300 B |
|  |  | 604.00 | 2.0527 | 0.1380 | 299 A→ 300 A |
|  | T_t_ | 638.95 | 1.9404 | 0.1226 | 298 B→ 299 B |
| DR2 | OS | 636.30 | 1.9485 | 0.0008 | 319 B→ 320 B (or 319 A→ 320 A) |
|  |  | 628.03 | 1.9742 | 0.1939 | 319 A→ 320 A (or 319 B→ 320 B) |
|  | T_t_ | 574.89 | 2.1567 | 0.0009 | 318 B→ 319 B |
| DR3 | OS | 649.44 | 1.9091 | 0.0674 | 319 A→ 320 A (or 319 A→ 320 A) |
|  |  | 649.00 | 1.9104 | 0.0162 | 319 B→ 320 B (or 319 A→ 320 A) |
|  | T_t_ | 593.65 | 2.0885 | 0.1656 | 318 B→ 319 B |
| DR4 | OS | 709.25 | 1.7481 | 0.0006 | 299 B→ 300 B |
|  |  | 607.01 | 2.0425 | 0.0013 | 299 A→ 300 A |
|  | T_t_ | 620.22 | 1.9990 | 0.0730 | 298 B→ 299 B |


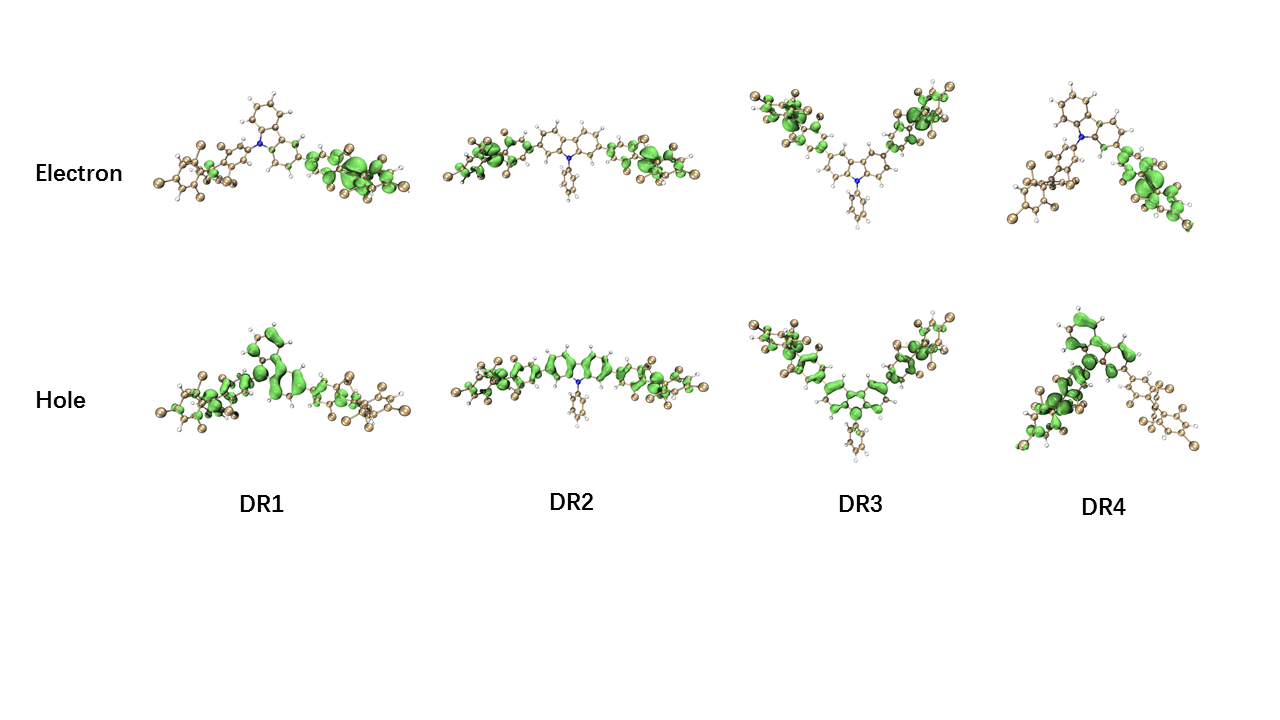


**Fig. S7** Hole-electron analysis of the lower charge transfer state transition based on OS state of DR1-DR4 (isovalue = 0.001 a.u.).


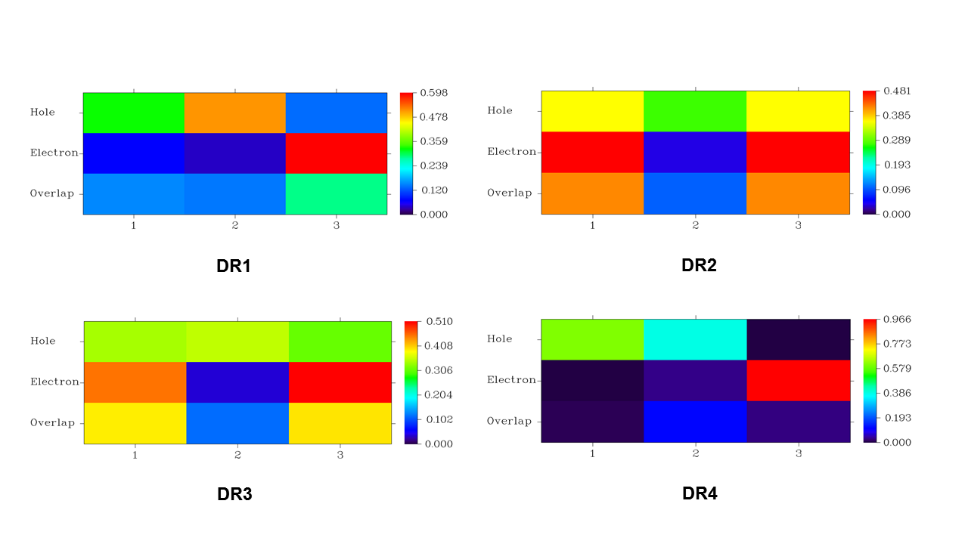


**Fig. S8** The fragment definition (up penals) and heat map of the second excited state transition based on OS state (down panels) of the four designed diradicals.

**Table S3.** The relevant index of low-lying charge transfer state of DR1-DR4 based on electron-hole analysis.

|  | DR1 | DR2 | DR3 | DR4 |
| --- | --- | --- | --- | --- |
| Sr(a.u.) | 0.518 | 0.870 | 0.832 | 0.099 |
| D(Å) | 7.812 | 0.461 | 2.091 | 9.425 |

**S2. Materials synthesis and characterisation**

**General:** All chemical agents and solvents, unless otherwise stated, were purchased from commercial suppliers and used directly without further purification. Tetrahydrofuran (THF) was distilled before used. Column chromatography was performed with silica gel (200-300 mesh).

The ^1^H nuclear magnetic resonance (NMR) spectra were recorded in (Methyl sulfoxide)-d6 (d6-DMSO) on a Bruker Avance-III 500 NMR spectrometer at ambient temperature. GC-MS mass spectra were recorded on a Thermo Fisher ITQ1100 mass spectrometer. MALDI-TOF mass spectra were recorded on a Bruker Autoflex speed TOF/TOF mass spectrometer with DCTB as a matrix. EPR spectra were recorded on a Bruker ELEXSYS-II E500 CW-EPR spectrometer. Thermal gravimetric analysis (TGA) curves were obtained on the Pyris1 TGA thermal analysis system at a heating rate of 20°C min^−1^ in a nitrogen atmosphere. Elemental analysis was conducted by an Elementar vario MICRO cube instrument. Ultraviolet-visible (UV-Vis) and photoluminescence (PL) spectra of the radicals were recorded on a Shimadzu UV-2550 spectrophotometer and a Shimadzu 5301PC spectrophotometer. The relative PLQYs were measured using a Shimadzu UV-2550 spectrophotometer and Edinburgh fluorescence spectrometer (FLS980). The fluorescence lifetimes were measured with FLS980.

The method for evaluating the photostability of luminescent radicals in solution is as follows: A solution (ca. 10^-5^ M, 3.0 mL) in 1-cm-optical-path-length quartz cells was bubbled with nitrogen, sealed and set at a Shimadzu 5301PC spectrophotometer. The intensity of luminescence at 654 nm (for DR1), 564 nm (for TTM), and 628 nm (for TTM-1Cz) were monitored exciting at 370 nm light (excitation slit was 20 nm, and shutter control was off). The logarithm of luminescence intensity versus time was plotted and a slope of the approximate line was estimated to be a rate of photolysis.

Single crystal X-ray diffraction data of DR1 was collected using a synchrotron X-ray source at the Shanghai Synchrotron Radiation Facility. The crystal structure was determined by direct methods, and further refined by the full matrix least squares method of F2 using the SHELX-97 and Olex-2.

Magnetic measurements were performed on a Quantum Design 6.5 Tesla SQUID-VSM system with a temperature range of 2 to 300 K and an applied field of 1000 Oe. Powder sample of DR1 with a weight of 5-10 mg was sealed in a plastic capsule. Magnetic moment was measured in the temperature range of 2 to 300 K. After correction of diamagnetic contributions from the sample, using tabulated constants, sample holder and paramagnetic contamination, the magnetic data were fitted with Bleaney-Bowers equation.^[13]^

The temperature-dependent PL spectra of the radicals were measured using a spectrometer (Ocean Optics QE65 Pro) and a Spectromag PT liquid helium free superconducting magneto-optical system (Oxford Instuments NanoScience) was used to provide different temperatures with 2~300 K and magnetic fields from 0 to 7 T.

The intermediate 2(HTTM), mono-radical TTM and TTM-1Cz are prepared according to our previous report. ^[14, 15]^

**Synthesis of 3.** Under argon atmosphere, HTTM (1.0 g, 1.8 mmol), 3-(4,4,5,5-tetraMethyl-1,3,2-dioxaborolan-2-yl)-carbazole (0.52 g, 1.8 mmol) and Pd(PPh_3_)_4_ (0.104 g, 0.09 mmol) were mixed in a 100 ml round-bottom flask containing toluene (12 ml), K_3_PO_4_ aqueous solution (8 ml, 2 M), and ethanol (4 ml), the mixture was refluxed at 90℃ for 48 h. After the reaction mixture cooling to room temperature, the mixture was extracted with dichloromethane. The organic layer was dried over anhydrous MgSO_4_. The crude product was purified by silica gel column chromatography (dichloromethane: petroleum =1:4) to obtain 3 as white powder with 44% yield. **GC-MS** (m/z): [M]^+^ Calcd for C_31_H_15_Cl_8_N：685.07, Found, 685.33. **^1^H NMR** (500 MHz, DMSO) δ 11.42 (s, 1H), 8.65 (d, J = 7.9 Hz, 1H), 8.26 (d, J = 7.9 Hz,1H), 7.99 (d, J = 2.0 Hz, 1H), 7.86-7.78 (m, 4H), 7.62 (dd, J = 4.4, 2.2 Hz, 2H), 7.55 (d, J=8.5 Hz,1H), 7.51 (d, J = 8.1 Hz,1H), 7.44-7.39 (m, 1H), 7.20 (t, J = 7.4 Hz, 1H), 6.73 (s, 1H).

**Synthesis of 4.** A mixture of TTM (0.50 g, 0.90 mmol), 3 (0.60 g, 0.90 mmol), anhydrous Cs_2_CO_3_ (0.90 g, 2.80 mmol) and DMF (30 ml) was stirred at 160℃ for 12 h under argon atmosphere and dark conditions. After the reaction mixture cooled to room temperature, it was poured into (1 M) hydrochloric acid solution; the precipitate was filtered and washed with water three times. The crude product was dissolved in dichloromethane and extracted with water and dichloromethane. The organic layer was dried over MgSO_4_, and evaporated under vacuum. The crude product was purified by silica gel column chromatography (dichloromethane: petroleum =1:3) to obtain 4 with 40% yield. **MALDI-TOF** (m/z): [M] Calcd for C_50_H_21_Cl_16_N:1202.93; Found, 1202.11. **^1^H NMR** (500 MHz, DMSO) δ 8.81 (s, 1H), 8.41 (d, J = 7.8 Hz, 1H), 8.01 (d, J = 13.7 Hz, 2H), 7.93 (d, J = 9.5 Hz, 1H), 7.84 (dd, J = 18.8, 7.9 Hz, 5H), 7.78 (s, 1H), 7.68 (s, 1H), 7.66 – 7.61 (m, 3H), 7.53 (d, J = 7.5 Hz, 1H), 7.48 (d, J = 8.7 Hz, 1H), 7.44 (d, J = 8.1 Hz, 1H), 7.40 – 7.37 (m, 1H), 6.82 (s, 1H), 6.74 (s, 1H).

**Synthesis of DR1.** Under argon atmosphere and in the dark, the KOtBu (0.31 g, 2.8 mmol) was added to the solution of 4 (0.30 g, 0.25 mmol) in dry THF and the solution acquired a light red colour. The solution was stirred for 12 h at room temperature, and then the 2,3,5,6-tetrachloro-p-benzoquinone (0.76 g, 3.1 mmol) was added. The solution was stirred for another 3 h. after the reaction finished, the solvent was removed under vacuum and further purified by silica gel column chromatography (dichloromethane: petroleum =1:2) to obtain DR1 as deep green solid 0.18g (61%). **MALDI-TOF** (m/z): [M] Calculated for C_50_H_19_Cl_16_N:1200.91; Found, 1200.29. **Elem. Anal.** Calculated for C_50_H_19_Cl_16_N: C 50.01, H 1.59, N 1.17. Found, C 50.16, H 1.76, N 1.21.

**S3.** **Crystallographic data of DR1**

**Table S4** X-Ray Crystallographic Data of DR1

| CCDC | 2252807 |
| --- | --- |
| Identification code  Empirical formula | DR1  C_50_H_19_Cl_16N_ |
| Formula weight | 1200.86 |
| Temperature/K | 273.15 |
| Crystal system | monoclinic |
| Space group | P21/c |
| a/Å | 25.3440(16) |
| b/Å | 8.4097(5) |
| c/Å | 26.7278(16) |
| α/° | 90 |
| β/° | 108.058(2) |
| γ/° | 90 |
| Volume/Å^3^ | 5416.0(6) |
| Z | 4 |
| ρcalcg/cm^3^ | 1.473 |
| μ/mm^‑1^ | 0.778 |
| F(000) | 2392.0 |
| Crystal size/mm^3^ | 0.2 × 0.05 × 0.05 |
| Radiation | Synchrotron (λ = 0.68878) |
| 2Θ range for data collection/° | 3.936 to 46.796 |
| Index ranges | -29<=h<=29, -9<=k<=9, -30<=l<=30 |
| Reflections collected | 49925 |
| Independent reflections | 8638 [R_int_= 0.0979, R_sigma_= 0.0680] |
| Data/restraints/parameters | 8638/3450/656 |
| Goodness-of-fit on F^2^ | 1.054 |
| Final R indexes [I>=2σ (I)] | R_1_ = 0.1400, wR_2_ = 0.3328 |
| Final R indexes [all data] | R_1_= 0.1516, wR_2_ = 0.3381 |
| Largest diff. peak/hole / e Å^-3^ | 1.70/-1.21 |

**S4. Magnetic susceptibility measurements of DR1**


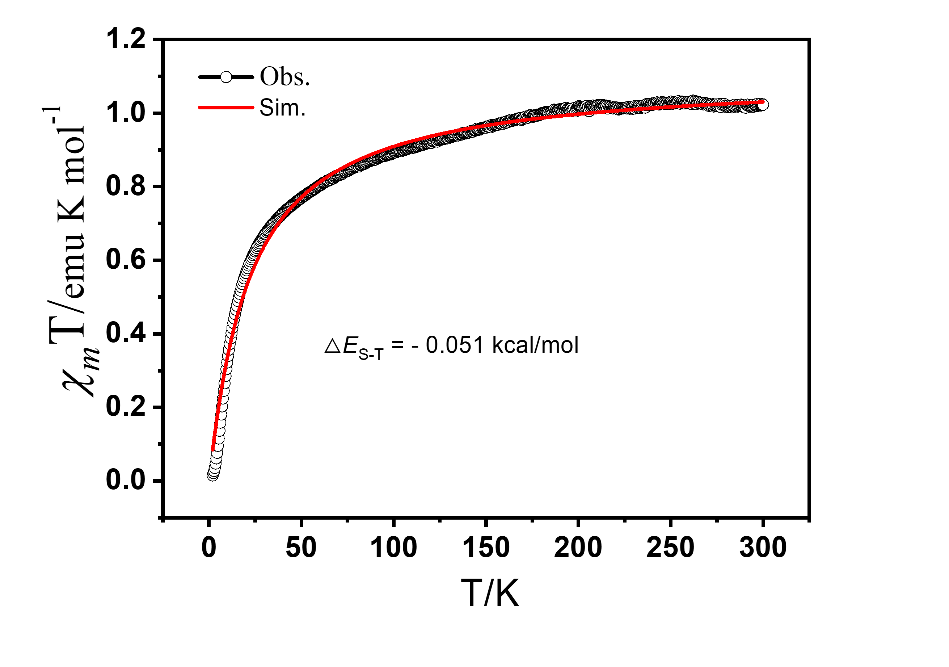


**Fig. S9** *χ_m_* *T - T* curve (circle) in the SQUID measurements for the powder of DR1 and the fitting plot obtained by the Bleaney-Bowers equation (red line).

**S5. Electron paramagnetic resonance (EPR) spectra of DR1**


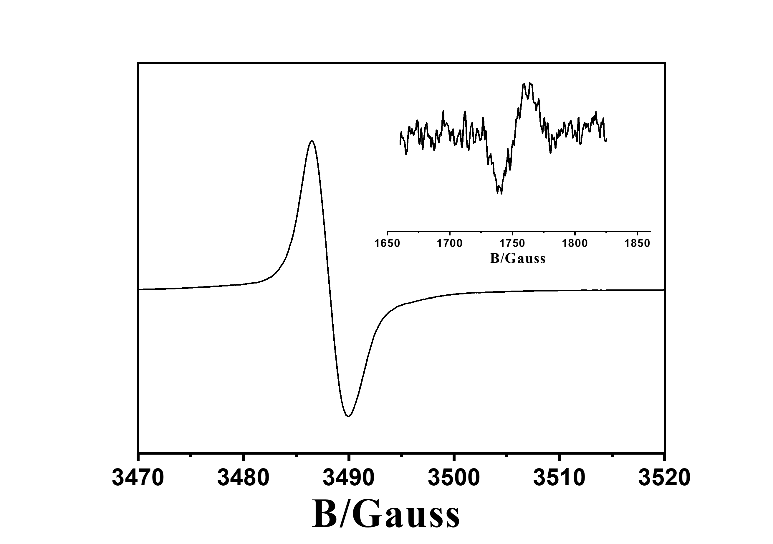


**Fig. S10** EPR spectra of **DR1** (1 wt%) in PMMA measured at 77 K; the inset shows the Δm_s_ = ±2 resonance.

**S6. Summary of photophysical properties for TTM, TTM-1Cz and DR1 in different solvents**


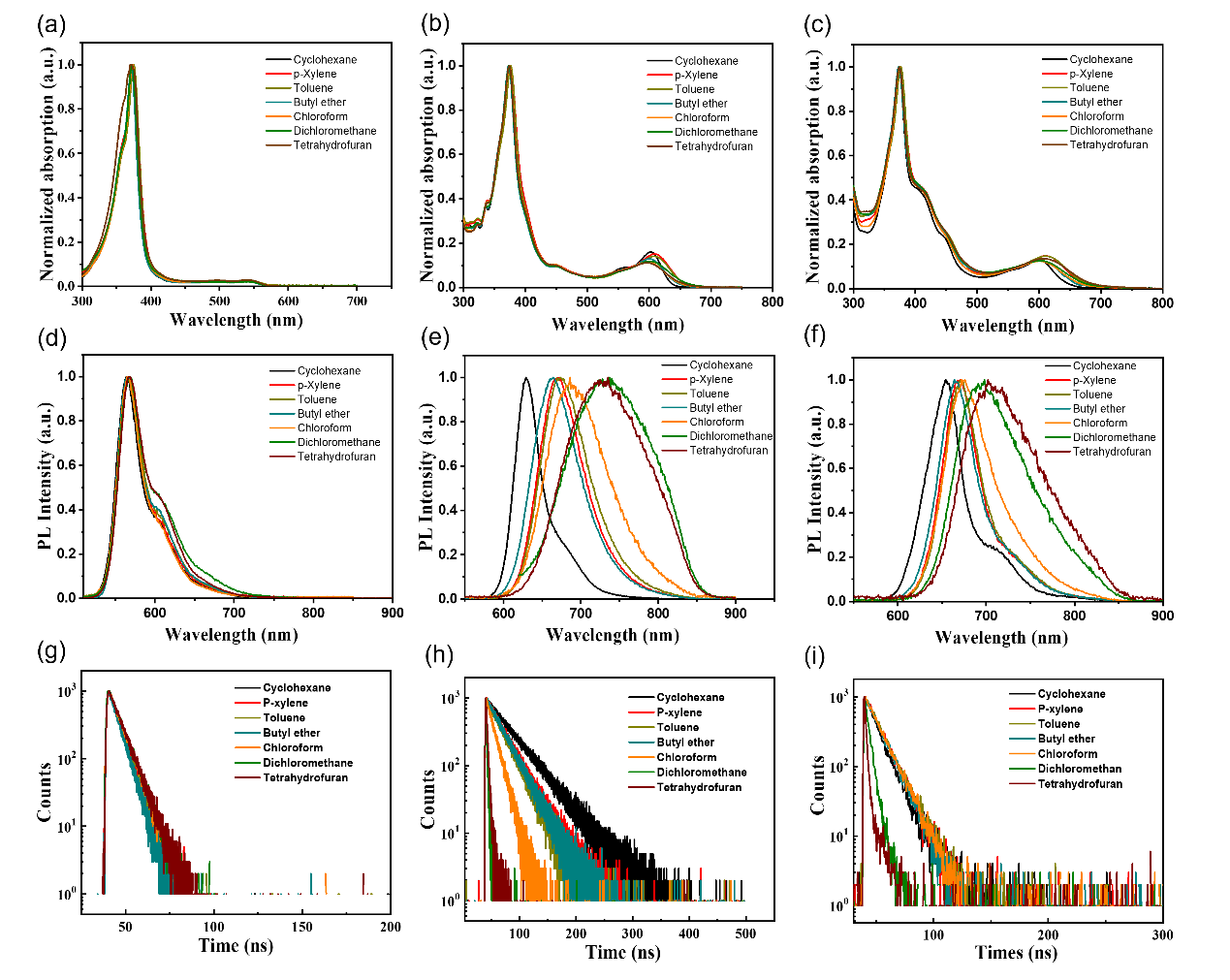


**Fig. S11** Normalised absorption, PL and transient PL decay spectra (excitation at 375 nm) of TTM (a, d, g), TTM-1Cz (b, e, h) and DR1 (c, f, i) in various solvents (10^-5^ M) at room temperature.

**Table S5.** Photophysical properties of TTM, TTM-1Cz and DR1 in various solvents.

| Radical | Solvent | ***λ***_abs_  (nm) | ***λ***_PL_ (nm) | τ  (ns) | PLQY  (%) | k_r_  (×10^7^ s^-1^) | k_nr_  (×10^7^ s^-1^) |
| --- | --- | --- | --- | --- | --- | --- | --- |
|  | Cyclohexane | 540 | 564 | 5.6 | 2.0 | 0.35 | 17.5 |
|  | p-Xylene | 543 | 567 | 5.7 | 2.4 | 0.42 | 17.1 |
|  | Toluene | 542 | 565 | 5.8 | 2.5 | 0.43 | 16.8 |
|  | Butyl ether | 541 | 565 | 5.9 | 2.4 | 0.41 | 16.5 |
| TTM | Chloroform | 541 | 566 | 6.0 | 2.6 | 0.43 | 16.2 |
|  | Tetrahyrofuran | 541 | 569 | 6.0 | 1.8 | 0.33 | 16.3 |
|  | Dichloromethane | 542 | 568 | 6.3 | 2.0 | 0.29 | 15.6 |
|  | Cyclohexane | 603 | 628 | 41.3 | 53.0 | 1.28 | 1.13 |
|  | p-Xylene | 609 | 670 | 28.7 | 23.0 | 0.80 | 2.68 |
|  | Toluene | 609 | 673 | 25.4 | 18.5 | 0.73 | 3.21 |
|  | Butyl ether | 602 | 663 | 26.9 | 23.0 | 0.85 | 2.86 |
| TTM-1Cz | Chloroform | 600 | 687 | 13.2 | 5.0 | 0.38 | 7.20 |
|  | Tetrahyrofuran | 603 | 735 | 1.3 | - | - | - |
|  | Dichloromethane | 599 | 728 | 1.6 | - | 1.28 | 1.13 |
|  | Cyclohexane | 600 | 654 | 10.6 | 16.0 | 1.51 | 7.92 |
|  | p-Xylene | 610 | 670 | 12.3 | 22.0 | 1.79 | 6.34 |
|  | Toluene | 610 | 672 | 12.7 | 25.0 | 1.97 | 5.91 |
| DR1 | Butyl ether | 602 | 665 | 12.1 | 20.0 | 1.65 | 6.61 |
|  | Chloroform | 602 | 675 | 12.5 | 19.0 | 1.52 | 6.48 |
|  | Tetrahyrofuran | 609 | 705 | 3.9 | 2.6 | 0.66 | 2.49 |
|  | Dichloromethane | 602 | 694 | 2.6 | 4.2 | 0.16 | 3.68 |

The radiative rate constants (k_r_) and non-radiative rate constants (k_nr_) of radicals were calculated using basic photophysical equations (1) and (2)

PLQY= *k_r_*/ (*k_r_* +*k_nr_*) (1)

τ=1/ (*k_r_* +*k_nr_*) (2)

**S7. Excitation and emission spectrum of DR1 in cyclohexane**


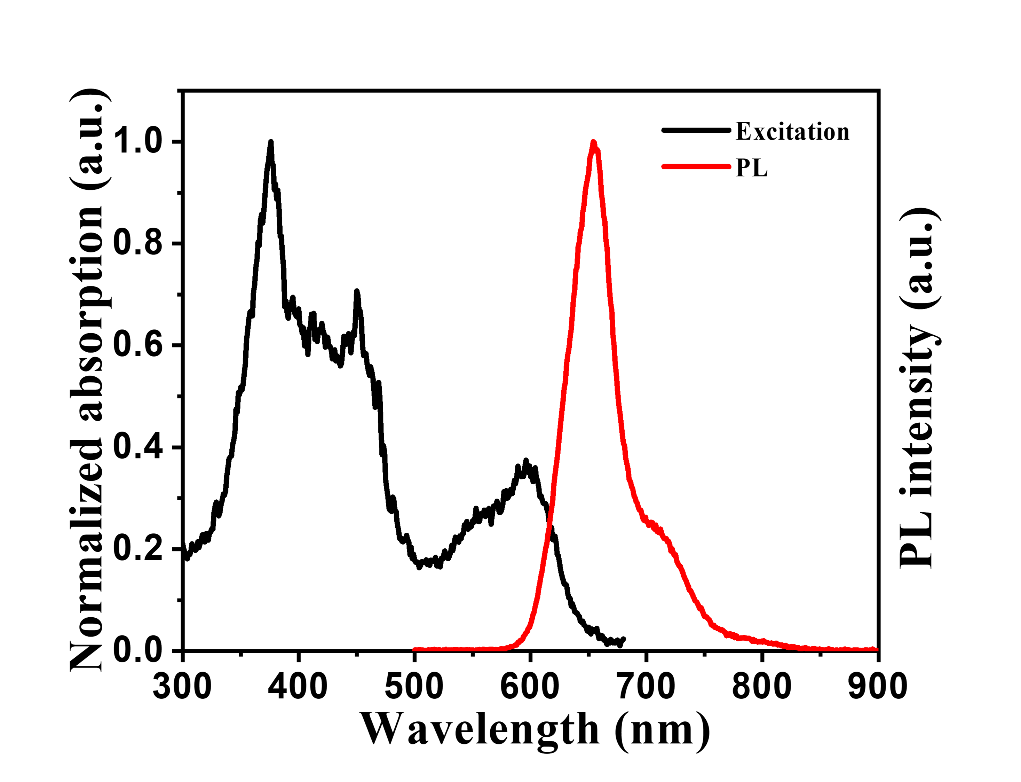


**Fig. S12** Excitation and emission spectrum of DR1 in cyclohexane at room temperature (monitored at 654 nm).

**S8. The influence of doping concentration and temperature of DR1 (in PMMA) on photoluminescence**


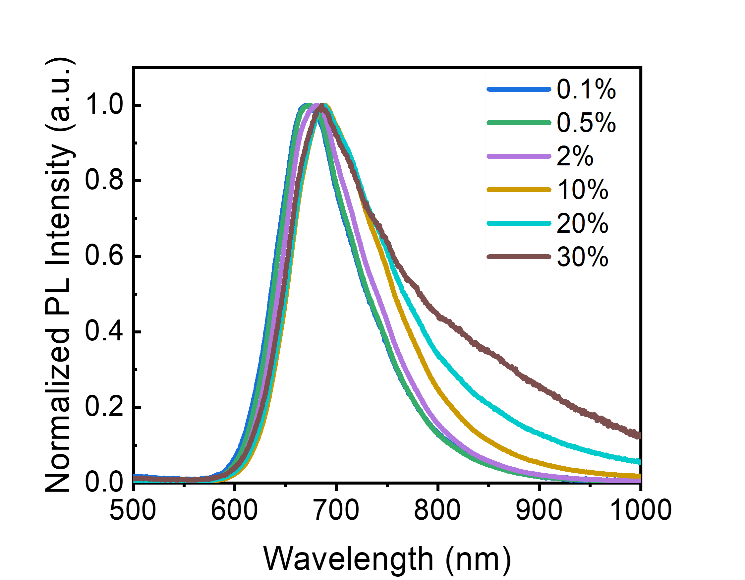


**Fig. S13** PL spectra of PMMA film with Different DR1 concentrations.

**Table S6** The Emission band and PLQYs of PMMA film with different DR1 concentrations.

| Wt.% | 0.1 | 0.5 | 2 | 10 | 20 | 30 |
| --- | --- | --- | --- | --- | --- | --- |
| Emission band (nm) | 671 | 673 | 680 | 689 | 690 | 689 |
| PLQY (%) | 5.2 | 14.1 | 13.3 | 1.2 | 0.24 | 0.12 |


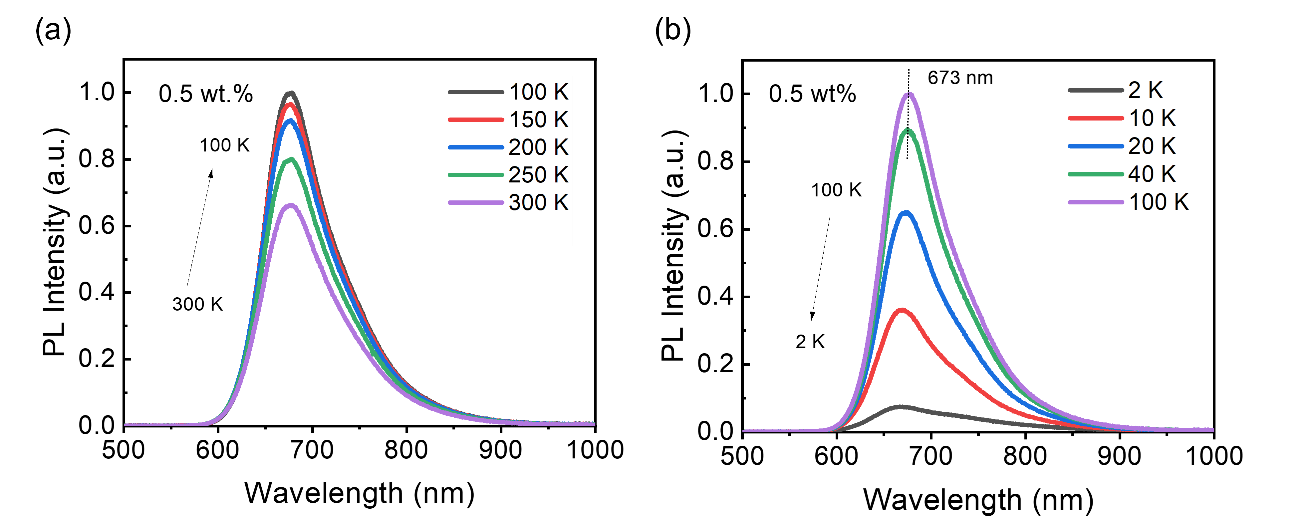


**Fig. S14** Temperature-dependent PL of DR1 (0.5 wt.%) doped in PMMA film.


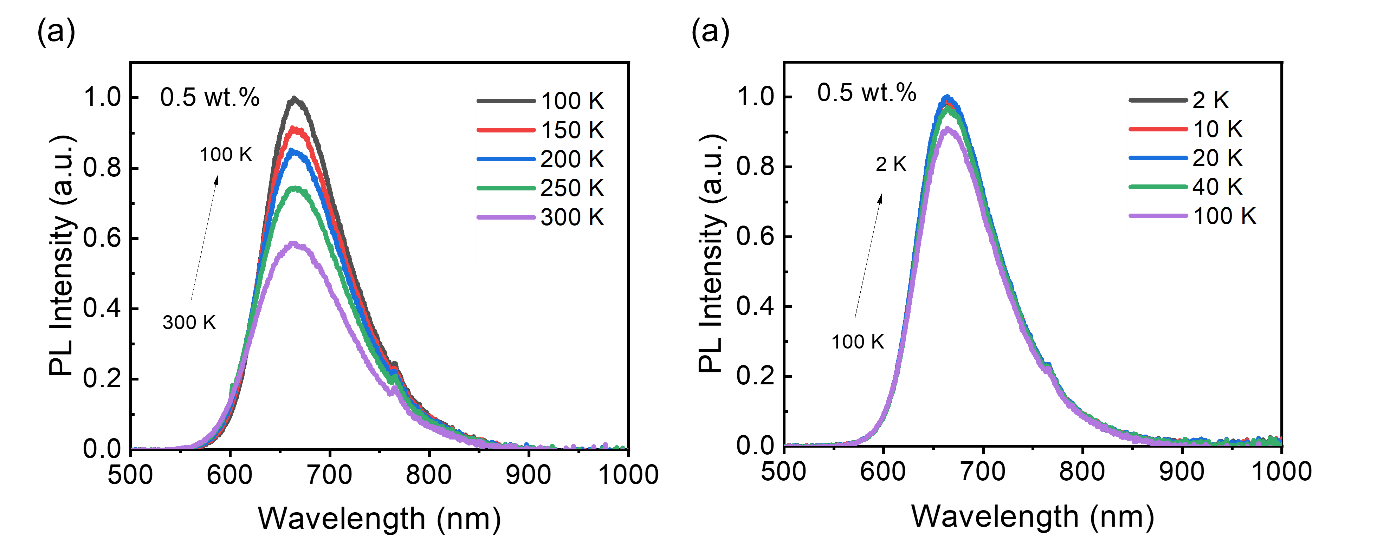


**Fig. S15** Temperature-dependent PL of TTM-1Cz (0.5 wt.%) doped in PMMA film.


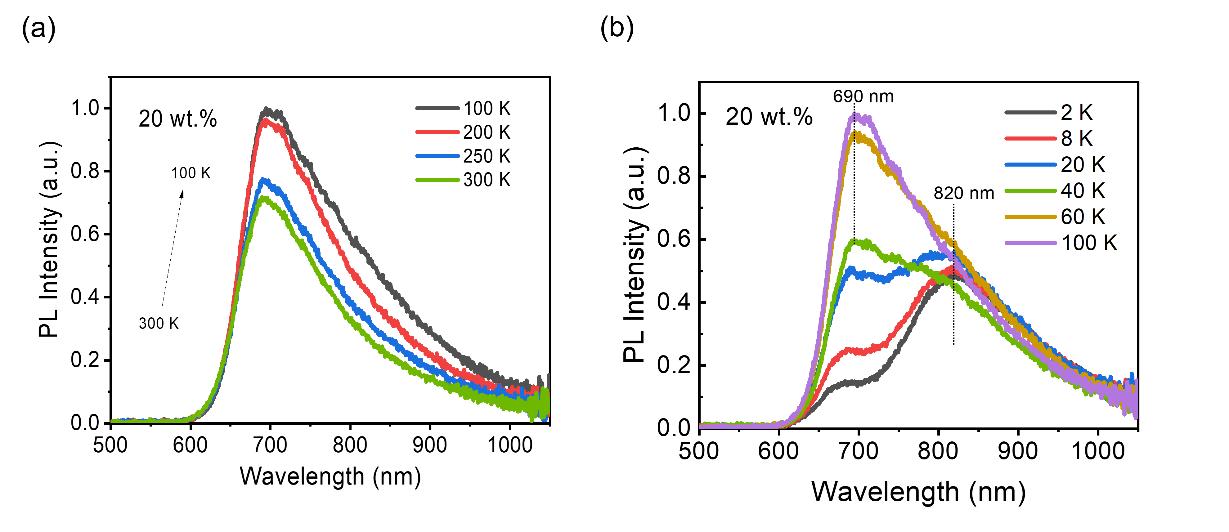


**Fig. S16** Temperature-dependent PL of DR1 (20 wt.%) doped in PMMA film.

**S9. Stablities of TTM, TTM-1Cz and DR1**

**
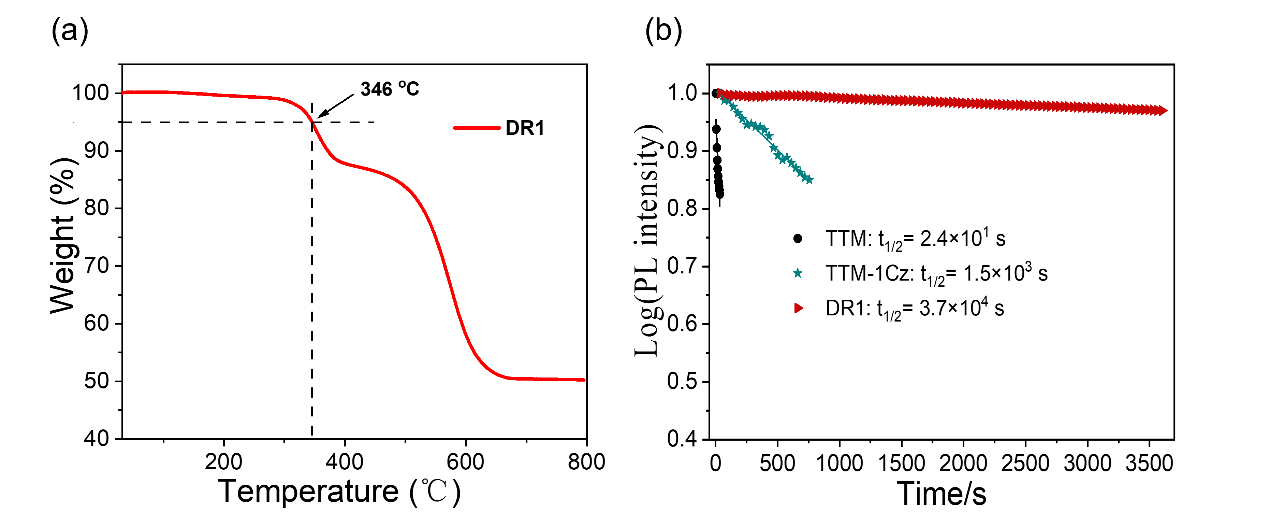
**

**Fig. S17** (a) TAG curve of DR1 under nitrogen flow. (b) Plots showing the PL intensity decay of radicals in cyclohexane under 375 nm continuous photoexcitation (Emission intensities are normalized).

**Table S7**. RSEs calculations of TTM, TTM-1Cz and DR1.

| Compd. | H(R)/a.u. | H(H·)/a.u. | H(R-H)/a.u. | BDE/kJ·mol^-1^ | RSE/KJ·mol^-1^ |
| --- | --- | --- | --- | --- | --- |
| TTM | -4869.135613 | -0.497912 | -4869.751848 | 310.644  (TTM-H) | 0.000 |
| TTM-1Cz | -4925.651396 | -0.497912 | -4926.267332 | 309.882  (TTM-1Cz-H) | 0.761 |
| DR1-OS | -9334.013261 | -0.497912 | -9334.629189 | 309.830  (DR1-H·) | 0.814 |
| DR1-Tr | -9334.013223 | -0.497912 | -9334.629189 | 309.935  (DR1-H·) | 0.709 |

*R represents the corresponding radical, R-H represents the precursor of the radical, and H▪ represents the hydrogen radical.

**Fig. S18** 3D sphere display of the steric descriptor of the buried volume (%*V*_Bur_). Steric maps of TTM, TTM-1Cz and DR1 radicals at radius of 6 Å (b), and at radius of 12 Å (c).

We calculated the RSE values of the DR1 for stabilizing one radical. As shown in Table S7, the RSE value of DR1-OS is larger than that of mono-radical TTM-1Cz, indicating the higher thermodynamic stability of the DR1. Besides, the RSE is used to characterize the thermodynamic stability of organic radicals. Generally, the kinetic stability is related to the lifetime (t_1/2_) of radicals that can reflect the photostability to some extent.^[16]^ The kinetic stability of radicals can be represented by describing the extent to which adjacent functional groups occupy the space around a radical center, which is quantified by the percentage of buried volumes (%V_Bur_).^[17,18]^ The DR1 radical’s steric is basically larger than that of TTM and TTM-1Cz radicals to form the steric protection for the central carbon atom (Fig. S18).

**S10. Electroluminescence properties of DR1-based OLED**

**
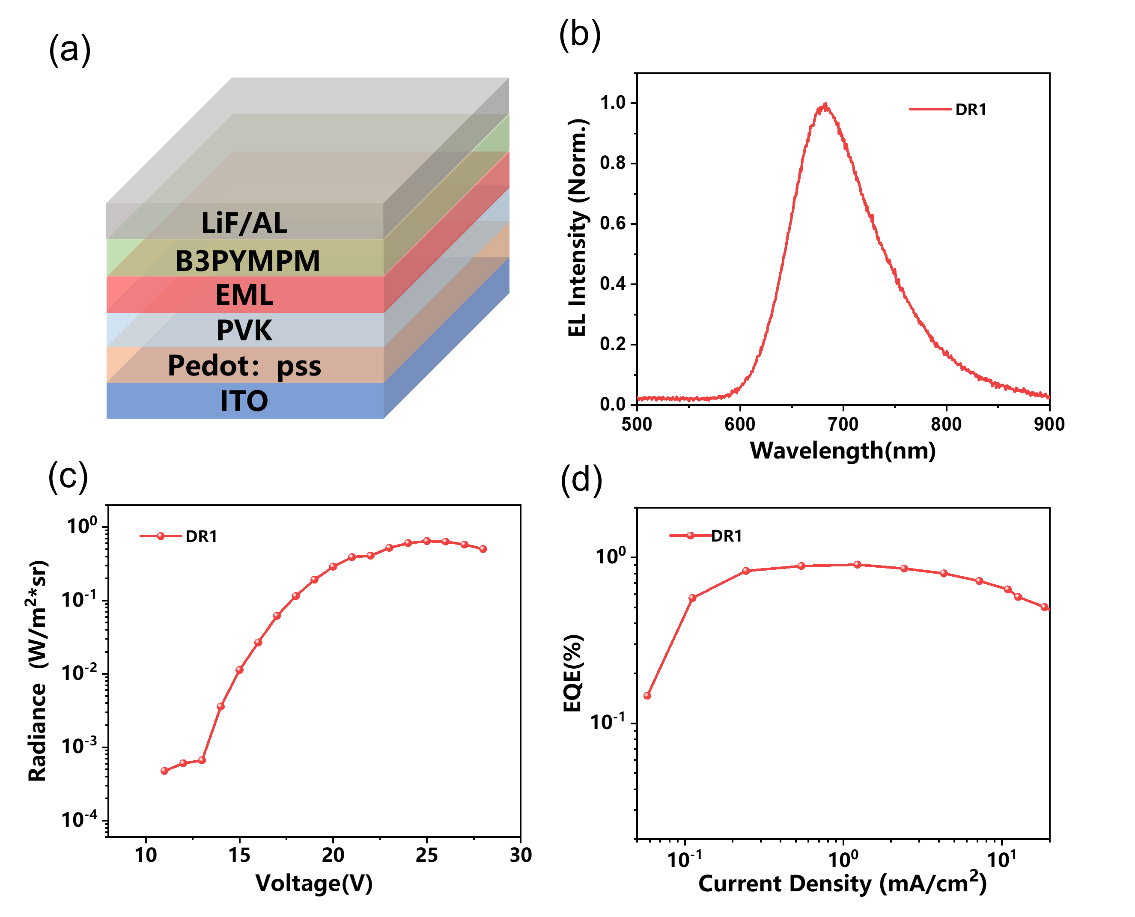
**

**Fig. S19** Optoelectronic properties of the OLED device with DR1 as the emitter. (a) Device structure diagram (b) EL spectra. (c) Radiance–voltage curves for the DR1 OLEDs. (d) EQE-current density.

**Solution-processing device fabrication:**

Indium tin-oxide (ITO; WF ∼ 4.8 eV) coated glass substrates were subsequently cleaned by sonication in acetone and 2-propanol for 10 minutes, followed by plasma 𝑂_2_ treatment for 10 minutes. PEDOT: PSS (Clevios CH4083, LumTech Taiwan, China) was spin-casted on top of the ITO under ambient conditions and annealed on a hot plate at 150°C for 10 minutes, forming a 40 nm-thick film. The substrates were then transferred to a nitrogen-filled glovebox to conduct the following solution processes 2. Poly(9-vinylcarbazone) (PVK, Sigma-Aldrich) as the hole injection layer (HIL) was spin-casted onto the PEDOT: PSS layer at the concentration of 15 mg/ml in chlorobenzene (Sigma-Aldrich) followed with an annealing process for 20 min at 120°C. The 40 nm-thick emitting layer (EML) of TPBi (Xi’an p-OLED Corp) doped with 0.5 wt.% of DR1 in chlorobenzene (20 mg/ml) was spin-coated on top of the PVK layer and baked on a hot plate at 90 °C for 10 min to remove any solvent present. The samples were then transferred to a vacuum deposition system. The 60 nm-thick electron-transporting layer (ETL) (B3PYMPM, Xi’an p-OLED Corp.), 1 nm-thick LiF (99.99%, Sigma-Aldrich) and 100 nm-thick aluminium were subsequently deposited by thermal evaporation under high vacuum (< 3×10−7 mbar)^2^.

**S11. Magneto-optical properties of DR1 and TTM-1Cz.**

**
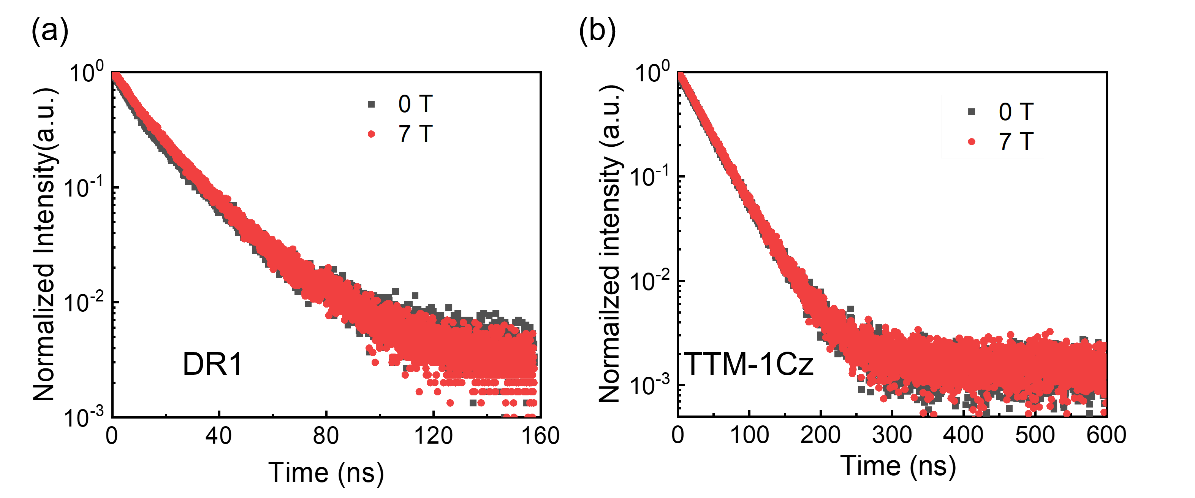
**

**Fig. S20** The transient PL decay spectra of TTM-1Cz (0.5 wt%) doped in PMMA film with and without a magnetic field of 7 T at 2 K.

**
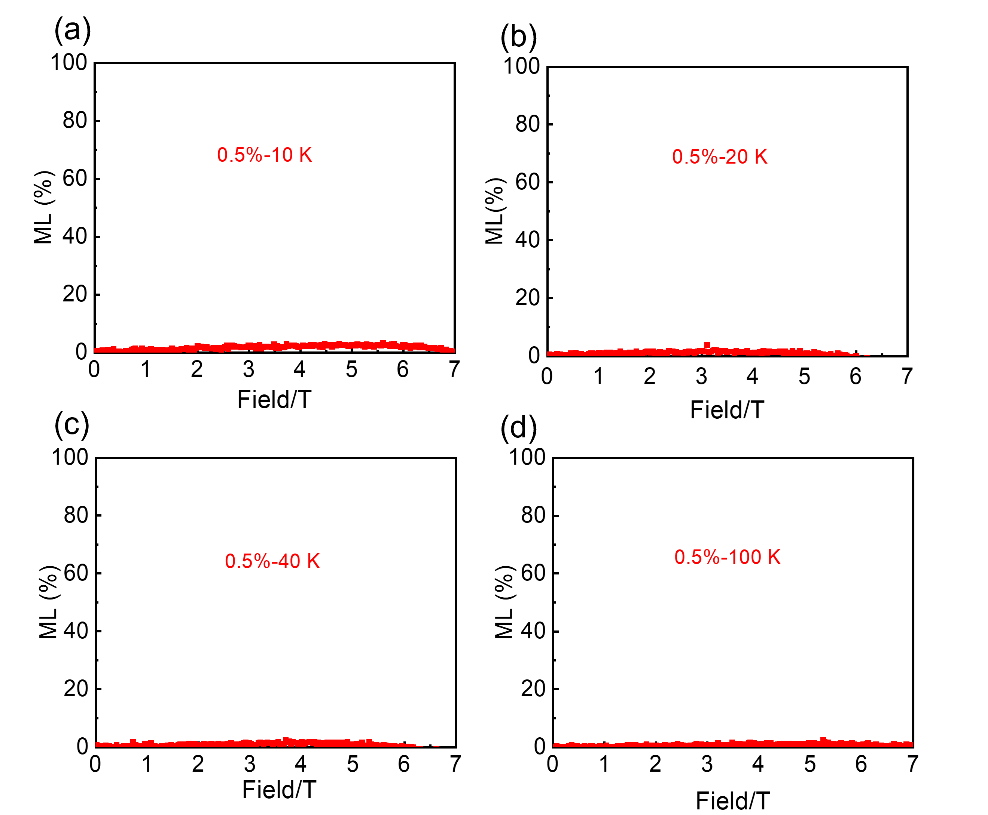
**

**Fig. S21** MLs of TTM-1Cz (0.5 wt%) doped in PMMA film in different temperature. (a) 8 K, (b) 20 K, (c) 40 K and (d) 100 K.

**
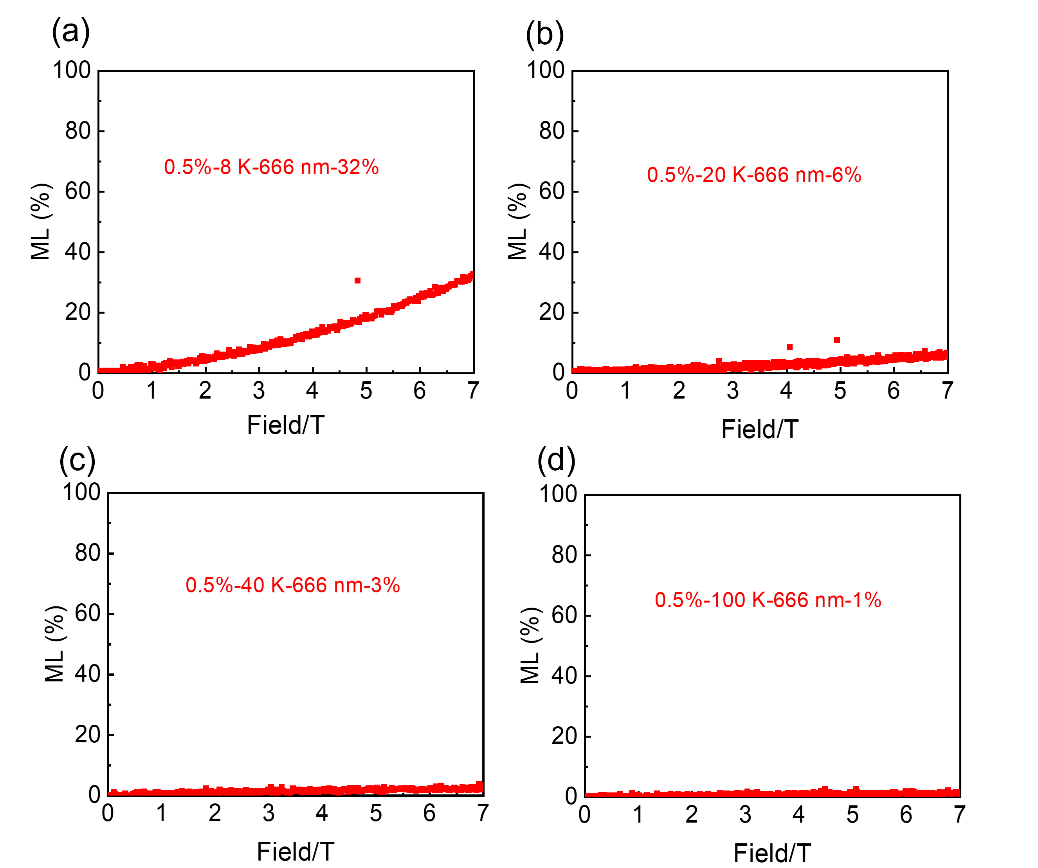
**

**Fig. S22** MLs of DR1(0.5 wt%) doped in PMMA film in different temperature. (a) 8 K, (b) 20 K, (c) 40 K and (d) 100 K.


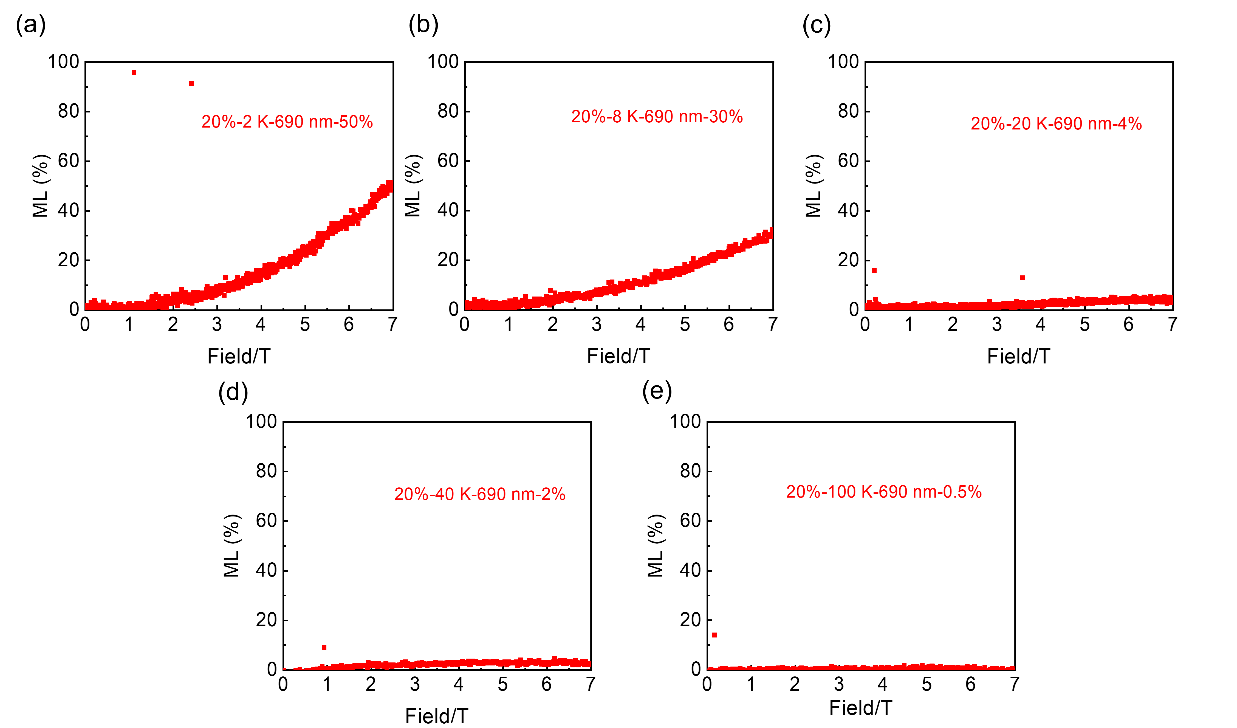


**Fig. S23** Temperature dependent ML of DR1 (20 wt.%) doped in PMMA film at 690 nm.


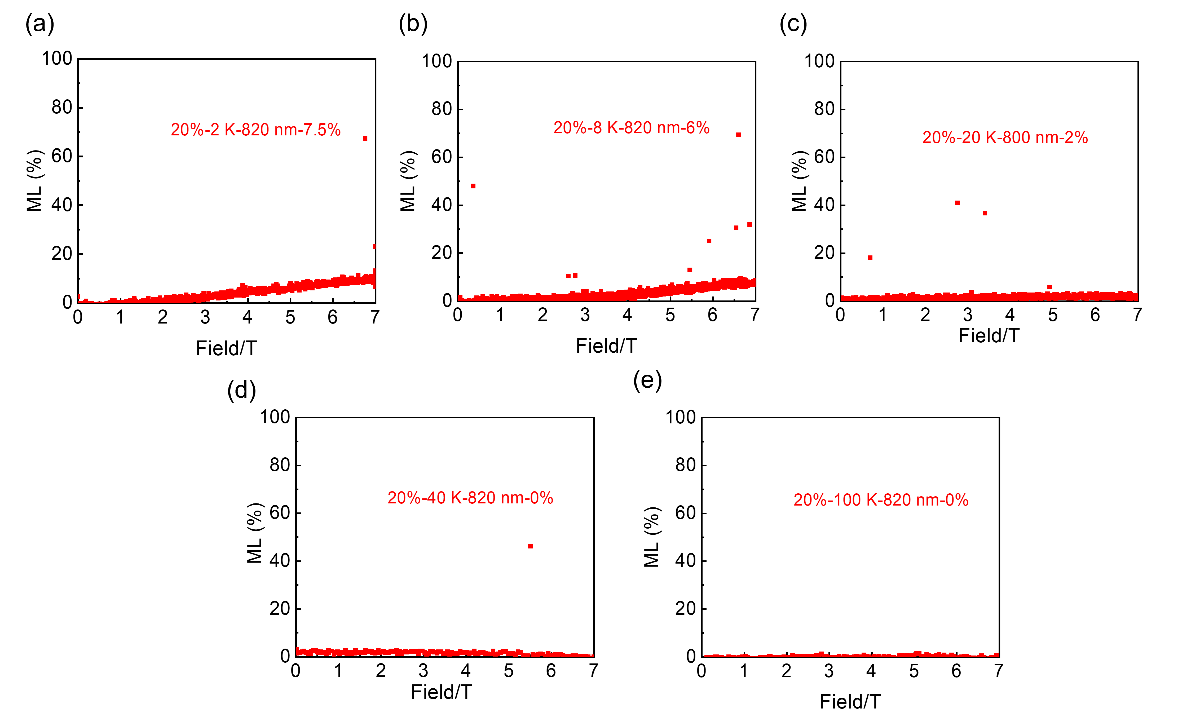


**Fig. S24** Temperature dependent ML of DR1 (20 wt.%) doped in PMMA film at 820 nm.

**References**

[1] M. J. Frisch, G. W. Trucks, H. B. Schlegel, G. E. Scuseria, M. A. Robb, J. R. Cheeseman, G. Scalmani, V. Barone, G. A. Petersson, H. Nakatsuji, X. Li, M. Caricato, A. V. Marenich, J. Bloino, B. G. Janesko, R. Gomperts, B. Mennucci, H. P. Hratchian, J. V. Ortiz, A. F. Izmaylov, J. L. Sonnenberg, D. Williams-Young, F. Ding, F. Lipparini, F. Egidi, J. Goings, B. Peng, A. Petrone, T. Henderson, D. Ranasinghe, V. G. Zakrzewski, J. Gao, N. Rega, G. Zheng, W. Liang, M. Hada, M. Ehara, K. Toyota, R. Fukuda, J. Hasegawa, M. Ishida, T. Nakajima, Y. Honda, O. Kitao, H. Nakai, T. Vreven, K. Throssell, J. A. Montgomery, Jr., J. E. Peralta, F. Ogliaro, M. J. Bearpark, J. J. Heyd, E. N. Brothers, K. N. Kudin, V. N. Staroverov, T. A. Keith, R. Kobayashi, J. Normand, K. Raghavachari, A. P. Rendell, J. C. Burant, S. S. Iyengar, J. Tomasi, M. Cossi, J. M. Millam, M. Klene, C. Adamo, R. Cammi, J. W. Ochterski, R. L. Martin, K. Morokuma, O. Farkas, J. B. Foresman, and D. J. Fox, Gaussian 16, Revision C.02, Wallingford CT, 2019.

[2] Stephens P. J.; Devlin, F.J.; Chabalowski, C.F. and Frisch M. J., *J. Phys. Chem*, 1994, 98: 11623.

[3] Frisch, M. J.; Pople, J. A.; Binkley, J. S., *J. Chem. Phys.* 1984, 80, 3265-3269.

[4] Yamaguchi, K., *Chem. Phys. Lett.*, 1975, 33, 330–335.

[5] Neese, F., The ORCA program system. *WIRES Comput. Mol. Sci.* 2012, 2, 73-78.

[6] Liu Z., Lu T., Chen Q., *Carbon*, 2020, 165, 461-467.

[7] Lu, T.; Chen, F., *J. Comput. Chem*. 2012, 33, 580-592.

[8] Humphrey, W., Dalke, A. and Schulten, K., *J. Molec. Graphics.* 1996, 14, 33-38.

[9] Shao, Y.; Head-Gordon, M.; Krylov, A. I., J. Chem. Phys. 2003, 118, 4807-4818.

[10] Becke, A. D. J. Chem. Phys. 1993, 98, 1372.

[11] Weigend, F.; Ahlrichs, R. Phys. Chem. Chem. Phys., 2005, 7, 3297-3305

[12] Abdurahman, A.; Wang, J.; Zhao, Y.; Li, P.; Shen, L.; Peng, Q., *Angew. Chem., Int. Ed*. 2023, 62, e202300772.

[13] Bleaney, B.; Bowers, K., *Proc. R. Soc. London, Ser. A* 1952, 214, 451- 465

[14] Peng, Q.; Obolda, A.; Zhang, M.; Li, F., *Angew.Chem.Int. Ed.* 2015, 54, 7091-7095;

[15] Abdurahman, A.; Hele, T. J. H.; Gu, Q.; Zhang. J.; Peng, Q.; Zhang, M.; Friend, R. H.; Li, F.; Evans, E. W., Nat. Mater. 2020, 19, 1224-1229.

[16] Feng, Z.; Tang, S.; Su, Y.; Wang, X., Chem. Soc. Rev. 2022, 51, 5930–5973

[17] Falivene, L.; Cao, Z.; Petta, A.; Serra, L.; Poater, A.; Oliva, R.; Scarano, V.; Cavallo, L., Nat. Chem. 2019, 11, 872-879.

[18] Wang, X.; Xue, P.; Zhou, C.; Zhang, Y.; Li, P.; Chen, R., J. Materi. Chem. C 2022, 10, 18343-18350.
